# *Arabidopsis* class I formins control membrane-originated actin polymerization at pollen tube tips

**DOI:** 10.1371/journal.pgen.1007789

**Published:** 2018-11-12

**Authors:** Yaxian Lan, Xiaonan Liu, Ying Fu, Shanjin Huang

**Affiliations:** 1 Center for Plant Biology, School of Life Sciences, Tsinghua University, Beijing, China; 2 Key Laboratory of Plant Physiology and Biochemistry, College of Biological Sciences, China Agricultural University, Beijing, China; UC-Riverside, UNITED STATES

## Abstract

A population of dynamic apical actin filaments is required for rapid polarized pollen tube growth. However, the cellular mechanisms driving their assembly remain incompletely understood. It was postulated that formin is a major player in nucleating apical actin assembly, but direct genetic and cytological evidence remains to be firmly established. Here we found that both *Arabidopsis* formin 3 (*AtFH3*) and formin 5 (*AtFH5*) are involved in the regulation of apical actin polymerization and actin array construction in pollen tubes, with AtFH3 playing a more dominant role. We found that both formins have plasma membrane (PM) localization signals but exhibit distinct PM localization patterns in the pollen tube, and loss of their function reduces the amount of apical actin filaments. Live-cell imaging revealed that the reduction in filamentous actin is very likely due to the decrease in filament elongation. Furthermore, we found that the rate of tip-directed vesicle transport is reduced and the pattern of apical vesicle accumulation is altered in formin loss-of-function mutant pollen tubes, which explains to some extent the reduction in pollen tube elongation. Thus, we provide direct genetic and cytological evidence showing that formin is an important player in nucleating actin assembly from the PM at pollen tube tips.

## Author summary

Actin polymerization has been implicated in the regulation of rapid polarized pollen tube growth. The important role of actin polymerization is well appreciated, but the mechanisms that regulate rapid actin polymerization in pollen tubes remain incompletely understood. It was postulated that one of the major actin polymerization pathways in pollen tubes involves formin/profilin modules. However, direct genetic and cytological evidence is still required to support the role of formin in this framework. Using state-of-the-art live-cell imaging in combination with reverse genetic approaches, we demonstrate here that two class I formins, *Arabidopsis* formin 3 (*AtFH3*) and formin 5 (*AtFH5*), are involved in the regulation of apical actin polymerization and actin array construction in pollen tubes. In support of the role of AtFH3 and AtFH5 in regulating membrane-originated apical actin polymerization, we found that both of them are localized to the plasma membrane (PM) at pollen tube tips. Live-cell imaging revealed that the reduction in filamentous actin is very likely due to the decrease in elongation of actin filaments originating from the apical membrane. We also found that AtFH3 and AtFH5 exhibit distinct PM localization patterns in the pollen tube, suggesting that they might have distinct roles in regulating actin polymerization in pollen tubes. Our study provides direct genetic and cytological evidence that formins act as important players in regulating apical actin assembly in pollen tubes.

## Introduction

The pollen tube is the passage for two non-motile sperm cells and its proper growth is essential for successful reproduction in flowering plants [1–3]. Pollen tube growth is tightly regulated, and this raises many fascinating questions. Numerous studies suggest that the actin cytoskeleton is the core of the regulatory network of pollen tube growth, presumably by coordinating with various cellular events, such as the trafficking, docking and fusion of vesicles and the construction of the cell wall [4–7]. Actin filaments are arranged into distinct structures within different regions of growing pollen tubes, and these structures carry out distinct cellular functions [8–13]. Highly dynamic actin filaments within the apical region were demonstrated to be directly associated with the growth and turning of pollen tubes [14–16]. To date, however, we still have an incomplete understanding of how those apical actin filaments are constantly generated in pollen tubes.

An essential step of actin polymerization is actin nucleation, which is controlled by various actin nucleation factors in cells. Among the actin nucleation factors identified in the literature [17], the formins and the Arp2/3 complex are found in plants [18, 19]. The formin proteins are characterized by the presence of two formin homology (FH) domains, FH1 and FH2, which are capable of nucleating actin assembly from actin or actin bound to profilin [18, 20–22]. Plant formins are categorized into two classes, designated as class I and class II. Class I formins have a transmembrane (TM) domain at their N-terminus followed by the C-terminal FH1 and FH2 domains, whereas class II formins do not have an N-terminal TM domain but carry an N-terminal phosphatase and tensin-related (PTEN)-like domain besides the conserved FH1 and FH2 domains [18, 19, 22–24]. *In vitro* biochemical analyses showed that most plant formins have the characteristic formin-mediated actin nucleating and barbed end capping and elongating activities [25–31]. Some plant formins were shown to have actin filament bundling [27–29, 32, 33] and microtubule interacting activities [29, 32–34], though the details of the mechanisms underlying these properties may vary between the different proteins.

As important regulators of actin dynamics, the plant formins have been implicated in numerous physiological cellular processes, such as epidermal pavement cell morphogenesis [35], cell division [32], cytokinesis [25], cell-to-cell trafficking [36], and interaction with pathogens [37], as well as the response to auxin signaling [38]. In particular, the formins have been implicated in polarized root hair growth [39–42] and pollen tube growth [4, 31, 43]. Specifically, after characterizing the cellular functions of the *Arabidopsis* formin gene *AtFH5*, Cheung et al. [4] proposed that formin nucleates actin assembly from the membrane for the construction of the subapical actin structure. In line with this finding, a recent report showed that the pollen-specific *Lilium longiflorum* Formin 1 (LiFH1) is involved in constructing the actin fringe structure [43]. However, considering that LIFH1 also has actin filament-bundling activity and was proposed to nucleate actin filaments from the surface of LiFH1-localized vesicles, more work is needed to understand how the formins fit into the apical actin polymerization pathway in general. Nonetheless, it was proposed that actin assembly mediated by formin-profilin modules may be a major pathway for actin polymerization from the apical membrane in the pollen tube [44]. The notion of formin acting as the major player in nucleating actin assembly is actually consistent with the scenario in which actin monomers are predicted to be buffered by an equal amount of profilin in pollen [45–47]. Considering that multiple formin isovariants exist in pollen, it is important to carefully document their precise intracellular localization and dynamics, their mechanism of action and their functional coordination.

Here, we showed that two class I formins, AtFH3 and AtFH5, localize to endomembrane systems and the plasma membrane (PM). However, they have distinct PM distribution patterns: AtFH3 is localized evenly throughout the entire pollen tube while AtFH5 is concentrated at pollen tube tips. We demonstrated that both AtFH3 and AtFH5 are involved in the regulation of membrane-originated actin polymerization within the growth domain of the pollen tube and they have overlapping function in this aspect. Loss of function of *AtFH3* and *AtFH5* reduces the velocity of tip-directed vesicle transport and alters the apical vesicle accumulation pattern in the pollen tube, further supporting the active role of apical actin filaments in regulating vesicle traffic. Thus, we provide strong evidence that class I formins control membrane-originated actin polymerization to enable the construction of the apical actin structure in the pollen tube.

## Results

### Both *AtFH3* and *AtFH5* are involved in the regulation of polarized pollen tube growth, with AtFH3 playing a more dominant role

**Fig 1:**
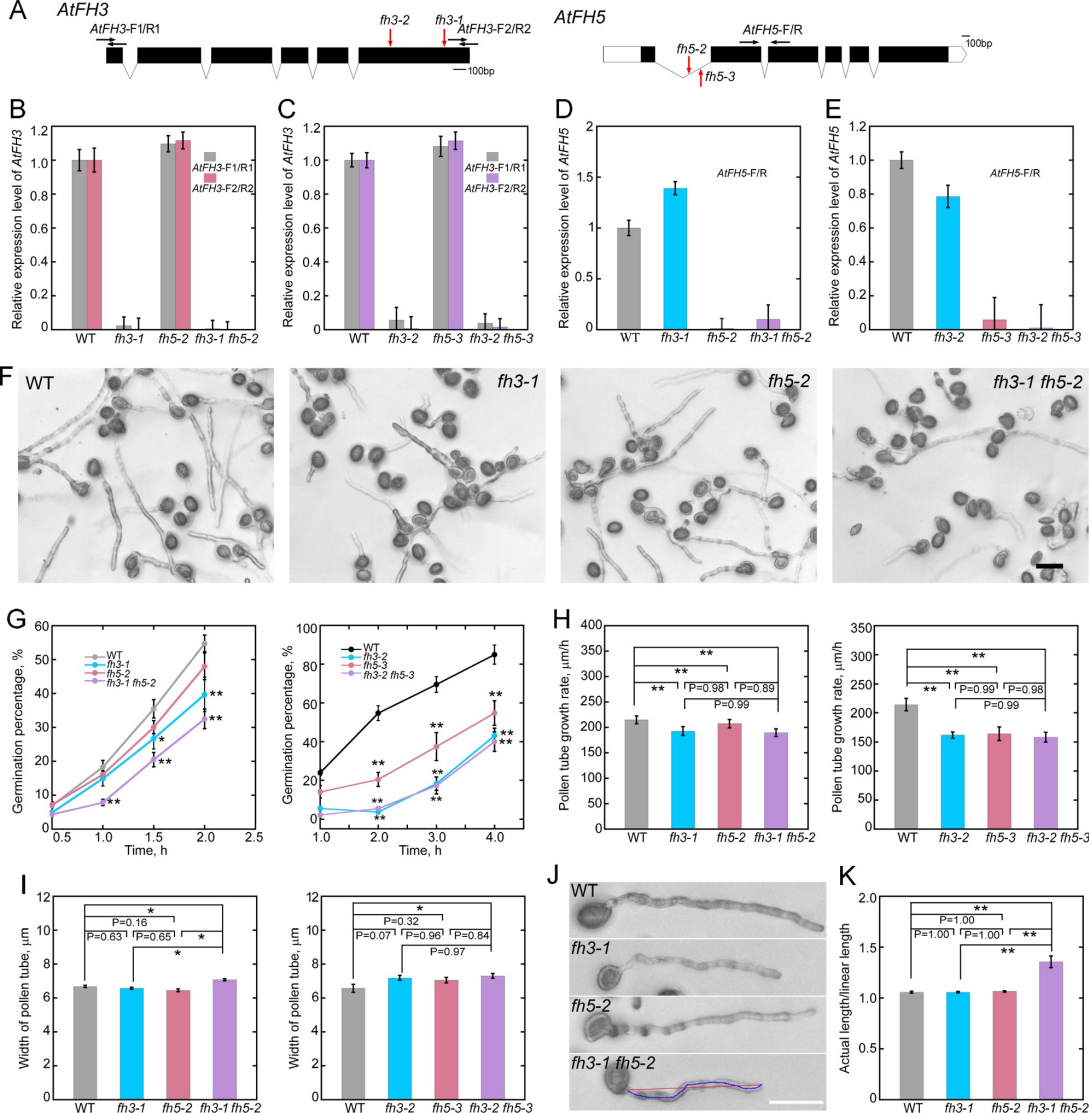
Both *AtFH3* and *AtFH5* are involved in the regulation of polarized pollen tube growth. (**A**) Physical structure of *AtFH3* (At4g15200) and *AtFH5* (At5g54650). Filled black boxes, lines and white boxes represent exons, introns and untranslated regions (UTRs), respectively. The T-DNA insertion alleles Salk_150350, CSHL_GT24923, Salk_044464 and Salk_152090 were designated as *fh3-1*, *fh3-2*, *fh5-2* and *fh5-3*, respectively. Red arrows indicate the position of T-DNA insertions. The black arrows indicate the position of primers used to analyze the transcript levels in pollen from WT and formin loss-of-function mutants. (**B**-**E**) qRT-PCR analysis of the amount of *AtFH3* (**B**, **C**) and *AtFH5* (**D, E**) transcripts in pollen derived from *fh3*, *fh5* and *fh3 fh5* mutant plants. Since T-DNA was inserted in the last exon of *AtFH3*, we used two primer pairs to detect the transcript level of *AtFH3* in *fh3-1* and *fh3-2* and to determine whether the truncated *AtFH3* transcript is transcribed in *fh3-1* and *fh3-2* mutant plants. *eIF4A* was used as the internal control. Data are presented as mean ± SE. (**F**) Micrographs of pollen derived from WT, *fh3-1*, *fh5-2* and *fh3-1 fh5-2* plants after 2 hours of incubation on standard germination medium are presented. Bar = 100 μm. (**G**) Quantification of pollen germination percentage in WT and formin loss-of-function mutants. The left panel is the plot of pollen germination percentage at different time points for WT, *fh3-1*, *fh5-2* and *fh3-1 fh5-2*, and the right panel is the plot of pollen germination percentage at different time points for WT, *fh3-2*, *fh5-3* and *fh3-2 fh5-3*. Data are presentend as mean ± SD, statistical comparisons were performed using Student’s *t*-test, *P < 0.05 and **P < 0.01. (**H**) Quantification of pollen tube growth rates. The left panel is the plot of pollen tube growth rates for WT, *fh3-1*, *fh5-2* and *fh3-1 fh5-2*, and the right panel is the plot of pollen tube growth rates for WT, *fh3-2*, *fh5-3* and *fh3-2 fh5-3*. Data are presented as mean ± SE, statistical comparisons were performed using ANOVA Post-Tukey, **P < 0.01. (**I**) Quantification of pollen tube width. The left panel is the plot of pollen tube width for WT, *fh3-1*, *fh5-2* and *fh3-1 fh5-2*, and the right panel is the plot of pollen tube width for WT, *fh3-2*, *fh5-3* and *fh3-2 fh5-3*. Data are presented as mean ± SE, statistical comparisons were performed using ANOVA Post-Tukey, *P < 0.05. (**J**) Micrographs of pollen tubes derived from WT and formin mutant plants. The blue and red lines in the bottom image indicate the actual length and linear length of the pollen tube, respectively. Bar = 100 μm. (**K**) Quantification of pollen tube morphology. The actual length was divided by the linear length and the values were plotted according to genotype to evaluate the morphology of the grown pollen tubes. Data are presented as mean ± SE, statistical comparisons were performed using ANOVA Post-Tukey, ** P < 0.01. More than 500 pollen tubes were measured for each genotype. Bar = 100 μm.

We previously showed that RNAi-mediated downregulation of *AtFH3* impairs the formation of shank-oriented longitudinal actin cables in the pollen tube [31]. However, more work is required to determine whether and how *AtFH3* may be involved in the regulation of actin polymerization within the apical region of the pollen tube. To better understand the mechanism of action of *AtFH3* in regulating actin polymerization in pollen cells, we sought to analyze stable T-DNA insertion mutants of *AtFH3*. In addition, considering that AtFH5 was previously shown to nucleate actin assembly from the plasma membrane for the construction of the subapical actin structure within the apical dome of the pollen tube [4], we also sought to determine whether there is functional coordination of *AtFH3* with *AtFH5* in regulating apical actin polymerization in pollen tubes. To this end, we analyzed T-DNA insertion mutants for *AtFH3* (*fh3-1* and *fh3-2*) and *AtFH5* (*fh5-2* and *fh5-3*) as well as the double mutants (*fh3-1 fh5-2; fh3-2 fh5-3*) (Fig 1A). The results showed that *fh3-1*, *fh3-2*, *fh5-2* and *fh5-3* are knockout alleles (Fig 1B–1E). We found that the pollen germination percentage was slightly but significantly reduced in pollen derived from *fh3-1*, *fh3-2*, *fh5-3*, *fh3-1 fh5-2* and *fh3-2 fh5-3* mutant plants (Fig 1F and 1G). In addition, we found that formin loss-of-function mutant pollen tubes grew significantly more slowly than WT pollen tubes (Fig 1H). Furthermore, we found that the width of pollen tubes was increased slightly but significantly in *fh3-1 fh5-2* and *fh3-2 fh5-3* double mutants compared to WT (Fig 1I). Interestingly, we found that growing *fh3-1 fh5-2* pollen tubes were more curved than WT, *fh3-1* or *fh5-2* pollen tubes (Fig 1J). This was supported by measurements showing that the ratio of the actual length of the pollen tube to the linear length was significantly increased in *fh3-1 fh5-2* pollen tubes (Fig 1K). Thus, these data suggest that *AtFH3* and *AtFH5* are required for normal polarized pollen tube growth, and AtFH3 has a more dominant role in this process.

### Loss of function of *AtFH3* and *AtFH5* reduces the amount of actin filaments in pollen and induces their disorganization within the apical region of pollen tubes

**Fig 2:**
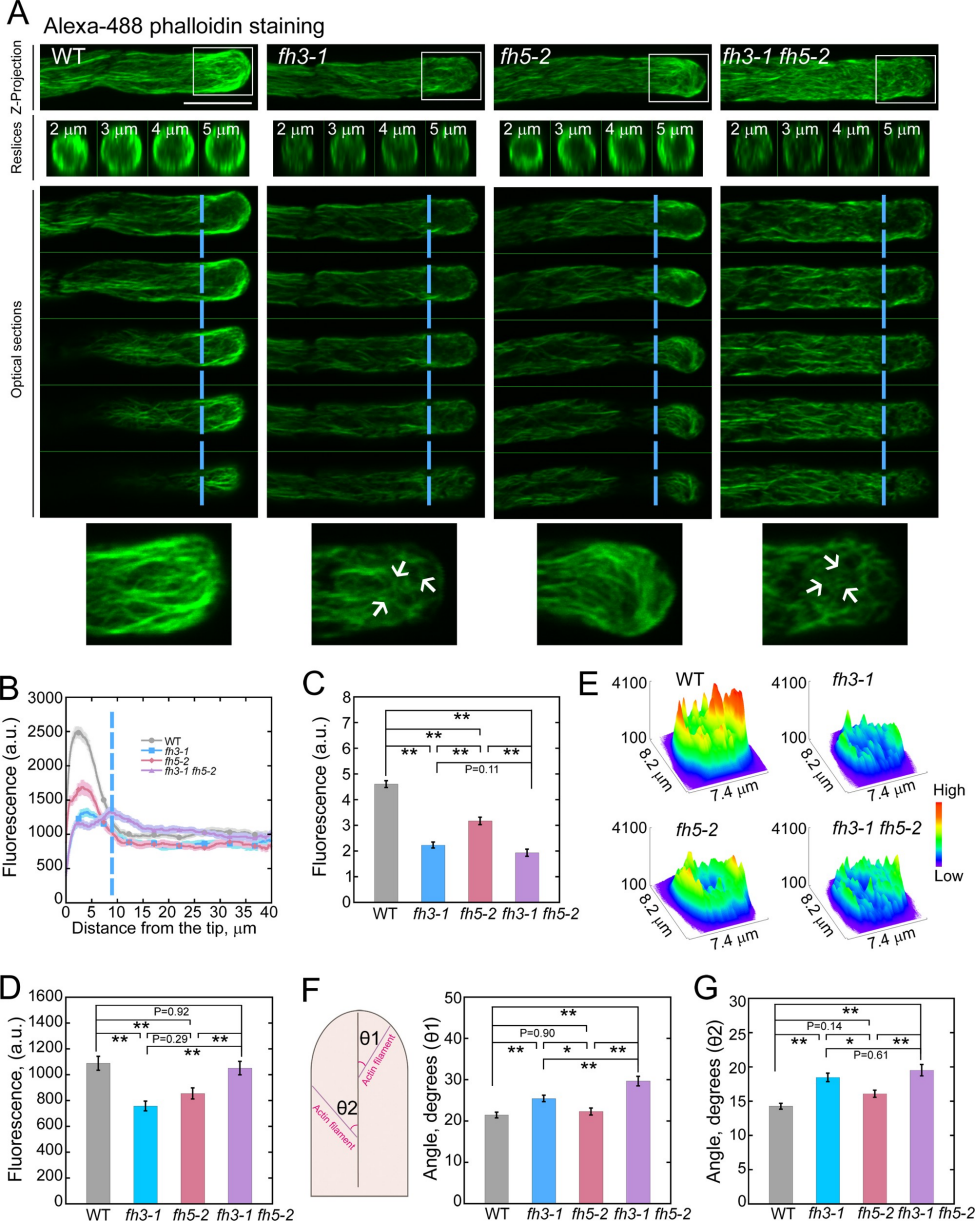
The amount of F-actin is reduced within the apical region of *fh3*, *fh5*, and *fh3 fh5* pollen tubes. (**A**) Micrographs of pollen tubes stained with Alexa-488 phalloidin. For each genotype, the upper panel shows the Z-projection images. The reslices of transverse sections of the boxed region are shown (their distance from the tip is indicated on the image). The longitudinal sections of the stained pollen tubes are also presented and the dashed blue lines indicate the base of the subapical region that was used to quantify the fluorescence intensity of the actin filaments. The bottom panels show enlarged images of the actin filament staining in the boxed region of the top panel. These images were projected from the 5 optical sections at the cortex (shown in the middle panels). White arrows indicate some short and fragmented actin bundles within the apical region of *fh3-1* and *fh3-1 fh5-2* pollen tubes. Bar = 10 μm. (**B**) Plot of the fluorescence intensity of actin filaments from the tip to the base of pollen tube. The dashed blue lines indicated the base of the subapical regions as shown in (**A**). (**C**) Quantification of the relative fluorescence intensity of actin filaments within the apical and subapical region (from the tip to the dashed blue line) of pollen tubes. Data are presented as mean ± SE, statistical comparisons were performed using ANOVA Post-Tukey, ** P < 0.01. (**D**) Quantification of the fluorescence intensity of actin filaments within the shank region of pollen tubes. Data were presented as mean ± SE, statistical comparisons were performed using ANOVA Post-Tukey, ** P < 0.01. (**E**) The 2D distribution of fluorescence pixel intensity of actin filaments within the apical region was generated using ImageJ software with a 3D interactive surface plot function. (**F**) Plot of the average angles formed between actin filaments and the pollen tube growth axis within the apical region of pollen tubes. The left panel is a schematic showing the measurement of the angles formed between actin filaments and the growth axis of the pollen tube. θ1 and θ2 represent the angles measured within apical region and shank region of the pollen tube, respectively. The right panel is the plot of average angles. Data represent mean ± SE. More than 200 apical actin filaments or bundles were measured from 10 pollen tubes for each genotype. Statistical comparisons were performed using ANOVA Post-Tukey, *P < 0.05, **P < 0.01. (**G**) Plot of the average angles (θ2) formed between actin filaments and the pollen tube growth axis within the shank region of pollen tubes. Data represent mean ± SE. More than 150 actin filaments were measured from 10 pollen tubes for each genotype. Statistical comparisons were performed using ANOVA Post-Tukey, *P < 0.05. **P < 0.01.

We next examined the organization of the actin cytoskeleton in WT and formin mutant pollen tubes using fluorescently labeled phalloidin. We found that the fluorescent signal from apical actin filaments was weaker in *fh3*, *fh5* and *fh3 fh5* mutant pollen tubes than in WT (Figs 2A and 2B and S1A). This is consistent with previously published work showing that both AtFH3 and AtFH5 are *bona fide* actin nucleation-promoting factors [25, 31]. The reduction in the level of filamentous actin was also noted in formin loss-of-function mutant pollen grains when they were compared to WT pollen grains (S2 Fig). It was previously proposed that within the pollen tube, actin filaments are arranged into two distinct arrays based on their origin [15]. Actin filaments originating from the apical membrane are organized into a unique apical actin structure which, in *Arabidopsis* pollen tubes, has its base 4–5 μm away from the tip [15]. Our results showed that the reduction in the level of actin filaments is very prominent within the region of the pollen tube that corresponds to the apical actin structure (Figs 2A and 2B and S1A). We evaluated the quantitative contribution of AtFH3 and/or AtFH5 to apical actin polymerization by measuring the amount of actin filaments within the region that is occupied by the apical actin structure. The results showed that loss of function of *AtFH3* causes a comparatively more severe reduction in the actin filament level than loss of function of *AtFH5* (Figs 2C and S1B). In addition, loss of function of both *AtFH3* and *AtFH5* caused an even more severe reduction in the actin filament level (Figs 2C and S1B), suggesting that AtFH3 and AtFH5 have overlapping function in regulating apical actin polymerization. We found that, although loss of function of *AtFH3* or *AtFH5* also causes reduction in the amount of actin filaments in the shank region, loss of function of both *AtFH3* and *AtFH5* does not have overt effect on the amount of actin filaments in the shank region of pollen tubes (Figs 2D and S1C). The reduction in the level of apical actin filaments was directly visualized by generating 3D plots of the 2D distribution of actin filament staining (Fig 2E). We found that, although the level of apical actin filaments in pollen tubes is not significantly different between single *AtFH3* loss-of-function mutants and *AtFH3* and *AtFH5* loss-of-function double mutants (Figs 2C and S1B), actin filaments appear more fragmented and disorganized within the apical region of pollen tubes from double mutants than from *fh3* single mutants (Figs 2A and S1A). To quantify the degree of actin filament disorganization, we measured the angles formed between the apical actin filaments and the growth axis of the pollen tube. We noticed that the angles were significantly higher in *fh3-1* and *fh3-1 fh5-2* mutant pollen tubes (Fig 2F), indicating that the apical actin filaments were relatively disorganized in these mutants. The increase in the angle is greater in *fh3-1 fh5-2* pollen tubes than in *fh3-1* pollen tubes (Fig 2F). The increase in the angle was also noticed for actin filaments within the shank region of formin loss-of-function mutant pollen tubes (Figs 2G and S1D). Thus, the data showed that both AtFH3 and AtFH5 are involved in the regulation of the polymerization and organization of apical actin filaments in the pollen tube, and AtFH3 has a more dominant role in this respect.

### Loss of function of *AtFH3* and/or *AtFH5* reduces the turnover rate of transport vesicles

**Fig 3:**
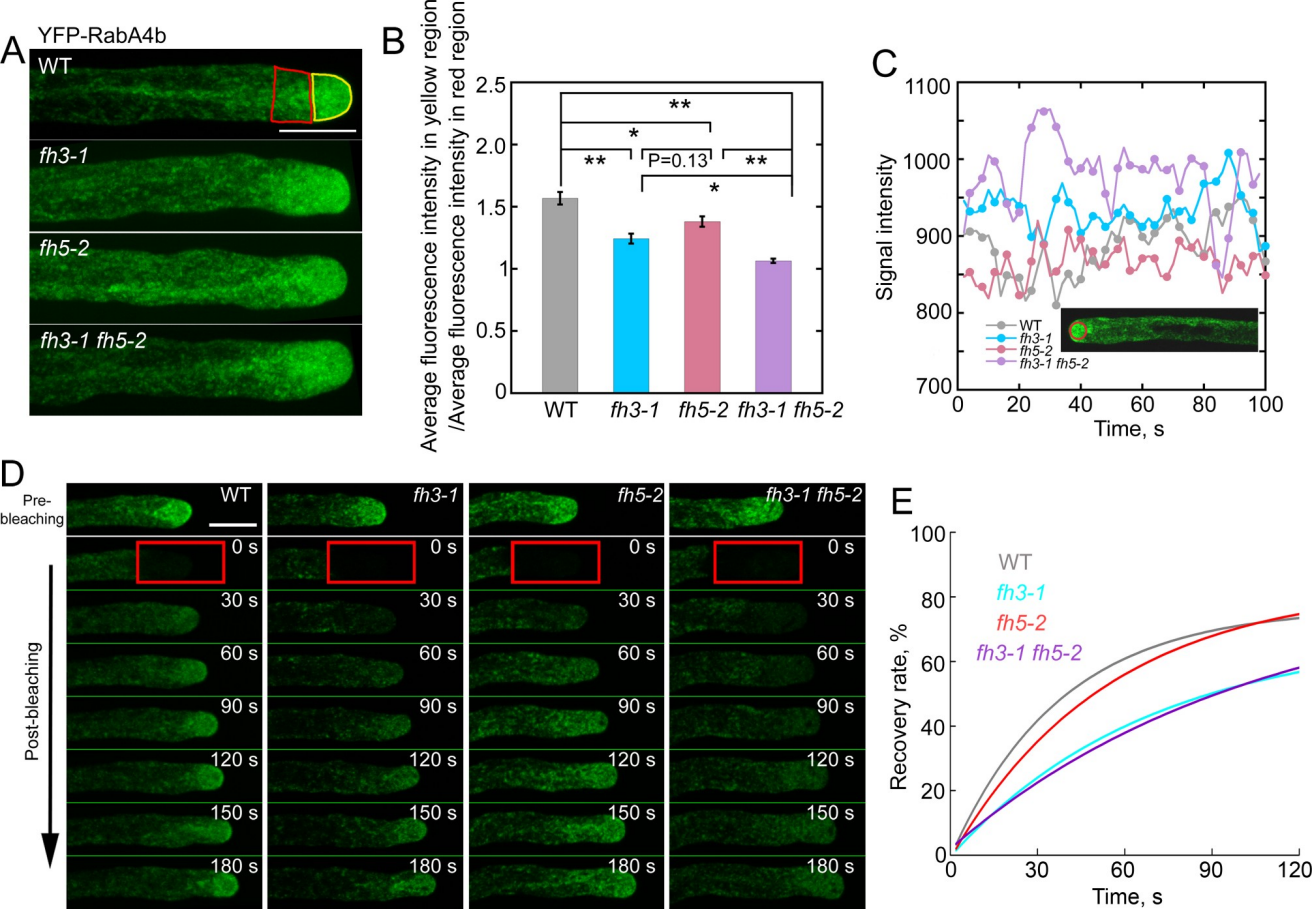
The apical vesicle accumulation pattern is altered and the turnover rate of vesicles is reduced in formin loss-of-function mutant pollen tubes. (**A**) Micrographs of pollen tubes expressing *Lat52*:*YFP-RabA4b*. The average pixel fluorescence intensity in the red and yellow boxed regions was determined in order to evaluate the vesicle accumulation pattern shown in (**B**). Bar = 10 μm. (**B**) Quantification of the accumulation pattern of RabA4b-positive vesicles in pollen tubes. The value of pixel fluorescence intensity in the red region was divided by that in the yellow region to yield a ratio to assess the extent of accumulation of RabA4b-positive vesicles. Data represent mean ± SE. Statistical comparisons were performed using ANOVA Post-Tukey, *P < 0.05, **P < 0.01. (**C**) Plot of YFP fluorescence pixel intensity as a function of the elapsed time. The value of fluorescence intensity within the red circle (shown in the inset pollen tube image) was measured every 2 s and plotted. The measurement was performed according to prevously published method [7]. (**D**) Micrographs of pollen tubes expressing *Lat52*:*YFP-RabA4b* before and after photobleaching. See the entire series in S1 Movie (WT), S2 Movie (*fh3-1*), S3 Movie (*fh5-2*) and S4 Movie (*fh3-1 fh5-2*). Red boxes indicate the photobleached regions. Bar = 10 μm. (**E**) Plot of YFP-RabA4b fluorescence versus recovery time. The values of YFP-RabA4b fluorescence were averaged and subjected to exponential curve fitting. The data were obtained from more than 20 pollen tubes and averaged.

To reveal how loss of function of *AtFH3* and/or *AtFH5* leads to the reduction in pollen tube growth, we examined the distribution of vesicles in pollen tubes. Transport vesicles were decorated with YFP-RabA4b as described previously [48]. We found that YFP-RabA4b-decorated transport vesicles accumulated in an inverted “V” cone shape in the WT pollen tube (Fig 3A). By contrast, the inverted “V” cone shape created by accumulation of apical vesicles is not very obvious in formin loss-of-function mutant pollen tubes (Fig 3A). Considering that the amount of apical actin filaments is reduced in formin loss-of-function mutant pollen tubes (Figs 2A–2C and 2E and S1A and S1B), this result is consistent with the notion that apical actin filaments spatially restrict vesicles within the apical region of the pollen tube [6, 15]. Surprisingly, we found that the region of vesicle accumulation was enlarged in *fh3-1 fh5-2* pollen tubes compared to WT pollen tubes (Fig 3A and 3B), which is presumably because the abnormally organized apical actin filaments cannot physically restrict the vesicles within the apical region. In addition, the fluorescence of vesicles is obviously brighter in *fh3-1 fh5-2* pollen tubes than in WT pollen tubes (Fig 3A and 3C), which is very likely because the backward movement of vesicles from the tip is severely reduced. To examine the dynamics of YFP-RabA4b-decorated vesicles, we used the technique of fluorescence recovery after photobleaching (FRAP). After bleaching the apical and subapical regions, we found that the recovery rate of vesicles is reduced in *fh3-1* and *fh3-1 fh5-2* mutant pollen tubes (Fig 3D and 3E and S1, S2 and S4 Movies). By comparison, the recovery rate of vesicles in *fh5-2* pollen tubes is only slightly slower than WT pollen tubes (Fig 3D and 3E and S1 and S3 Movies). The extent of the alteration in vesicle recovery rate within the apical and subapical regions of the pollen tube correlates well with the extent of the reduction in apical actin filaments (Figs 2A–2C and 2E and S1A and S1B). Thus, the results showed that loss of function of AtFH3 and/or AtFH5 alters the apical vesicle accumulation pattern and reduces the rate of vesicle turnover in the pollen tube.

### AtFH3 and AtFH5 localize to the plasma membrane (PM) and endomembrane system in the pollen tube

**Fig 4:**
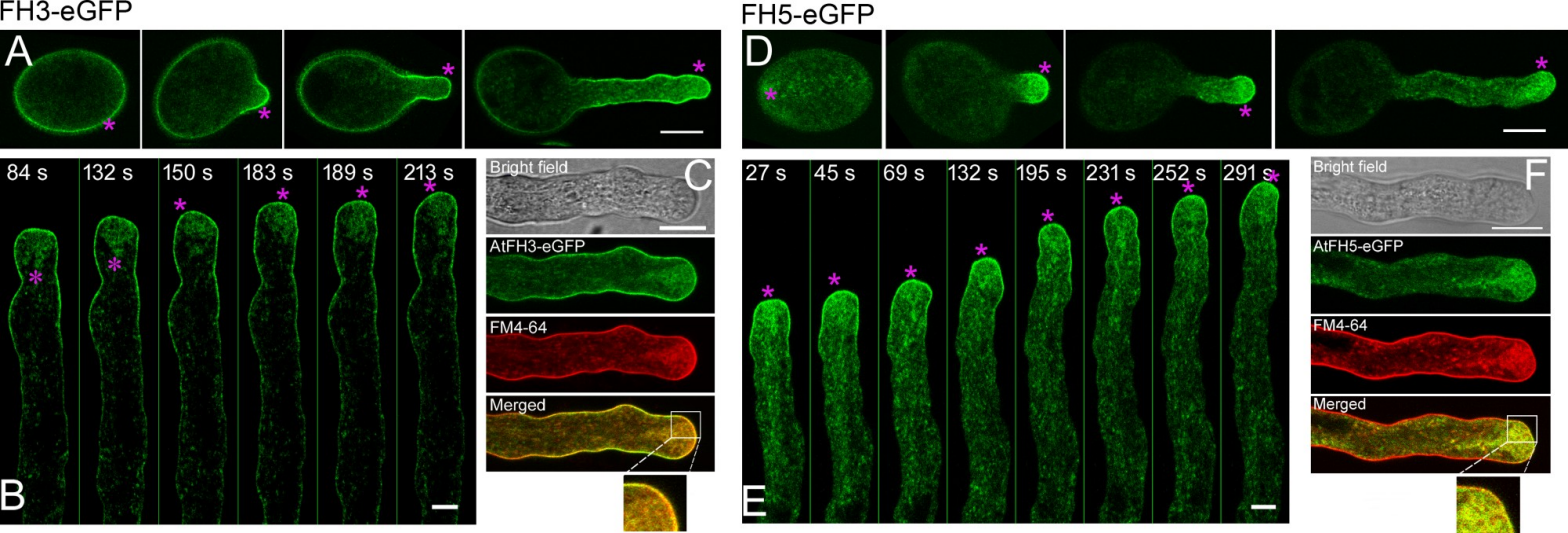
Intracellular localization of AtFH3 and AtFH5 in pollen tubes. (**A**) Distribution of AtFH3-eGFP in ungerminated and germinated pollen derived from *AtFH3pro*:*FH3-eGFP;fh3-1* plants. Medial optical sections are presented. The magenta asterisks indicate the localization of AtFH3-eGFP on the PM. Bar = 10 μm. (**B**) Time-lapse images of AtFH3-eGFP in the growing pollen tube. The magenta asterisks indicate the localiztion of AtFH3-eGFP on the PM and vesicles. Bar = 5 μm. (**C**) Co-localization of AtFH3-GFP with FM4-64-stained plasma membrane and endocytic vesicles. Bar = 10 μm. (**D**) Distribution of AtFH5-eGFP in ungerminated and germinated pollen derived from *AtFH5pro*:*FH5-eGFP;fh5-2* plants. Medial optical sections are presented. The magenta asterisks indicate the localization of AtFH5-eGFP on the PM. Bar = 10 μm. (**E**) Time-lapse images of AtFH5-eGFP in the growing pollen tube. The magenta asterisks indicate the localization of AtFH5-eGFP on the PM and vesicles. Bar = 5 μm. (**F**) Co-localization of AtFH5-GFP with FM4-64-stained plasma membrane and endocytic vesicles. Bar = 10 μm.

To determine the precise intracellular localization of AtFH3 and AtFH5, we generated green fluorescent protein (GFP) fusion constructs of AtFH3 and AtFH5 driven by their own promoters, *AtFH3pro*:*AtFH3-eGFP* and *AtFH5pro*:*AtFH5-eGFP*, and transformed them into *fh3-1* and *fh5-2* to generate the transgenic plants *AtFH3pro*:*AtFH3-eGFP;fh3-1* and *AtFH5pro*:*AtFH5-eGFP;fh5-2*, respectively. We found that transformation of those constructs rescued the defects in the amount and organization of apical and subapical actin filaments (S3 Fig), suggesting that the GFP fusion constructs are functional. Confocal microscopy revealed that both AtFH3-eGFP and AtFH5-eGFP form punctate structures in the cytoplasm of pollen grains and pollen tubes (Fig 4A, 4B, 4D and 4E). This suggests that the fusion proteins are associated with the endomembrane system, which is consistent with previous characterization of AtFH5 [4]. In addition, both AtFH3 and AtFH5 are able to localize to the PM but exhibit distinct patterns: AtFH3 is localized quite evenly on the PM along the entire pollen tube (Fig 4A and 4B and S5 Movie) while AtFH5 is concentrated on the PM within the apical dome of the tube (Fig 4D and 4E and S6 Movie). After plasmolysis, AtFH5-eGFP retained its association with apical membranes while AtFH3-eGFP was retained on the PM along the pollen tube (S4 Fig). AtFH3 and AtFH5 also had distinct localization patterns in ungerminated pollen grains, with AtFH3 exhibiting obvious PM localization (Fig 4A) whereas AtFH5 does not exhibit obvious PM localization (Fig 4D). The endomembrane and PM localization of AtFH3 and AtFH5 in pollen tubes were further confirmed by staining with FM4-64 dyes, which showed that AtFH3-eGFP and AtFH5-eGFP overlapped with FM4-64 dyes on the cell membrane and punctate structures within the cytoplasm (Fig 4C and 4F). The subcellular localization data are consistent with the presence of a transmembrane (TM) domain in AtFH3 and AtFH5 [4, 25, 31]. We found that depolymerization of the actin cytoskeleton (S5A Fig) does not prevent the PM and endomembrane targeting of AtFH3 and AtFH5 (S5B and S5C Fig). This suggests that their targeting to the PM and endomembranes in pollen tubes does not require their interaction with the actin cytoskeleton. Furthermore, we found that the N-terminus of AtFH3, which contains the signal peptide (SP) and TM, is sufficient for targeting of AtFH3 to the PM and endomembranes (S6A Fig). This is consistent with previous observations that the membrane localization of LiFH1 is determined by its N-terminus, which also contains the SP-TM domain [43]. Strikingly, we found that replacement of the TM domain of AtFH3 with that of AtFH5 endows AtFH3 with a PM localization pattern similar to that of AtFH5 (S6B Fig). Thus, our study suggests that both AtFH3 and AtFH5 are able to localize to the PM and endomembrane system, and the membrane localization pattern is dictated by their TM domains.

### Loss of function of *AtFH3* and *AtFH5* reduces the elongation rate of actin filaments originating from the apical membrane

**Fig 5:**
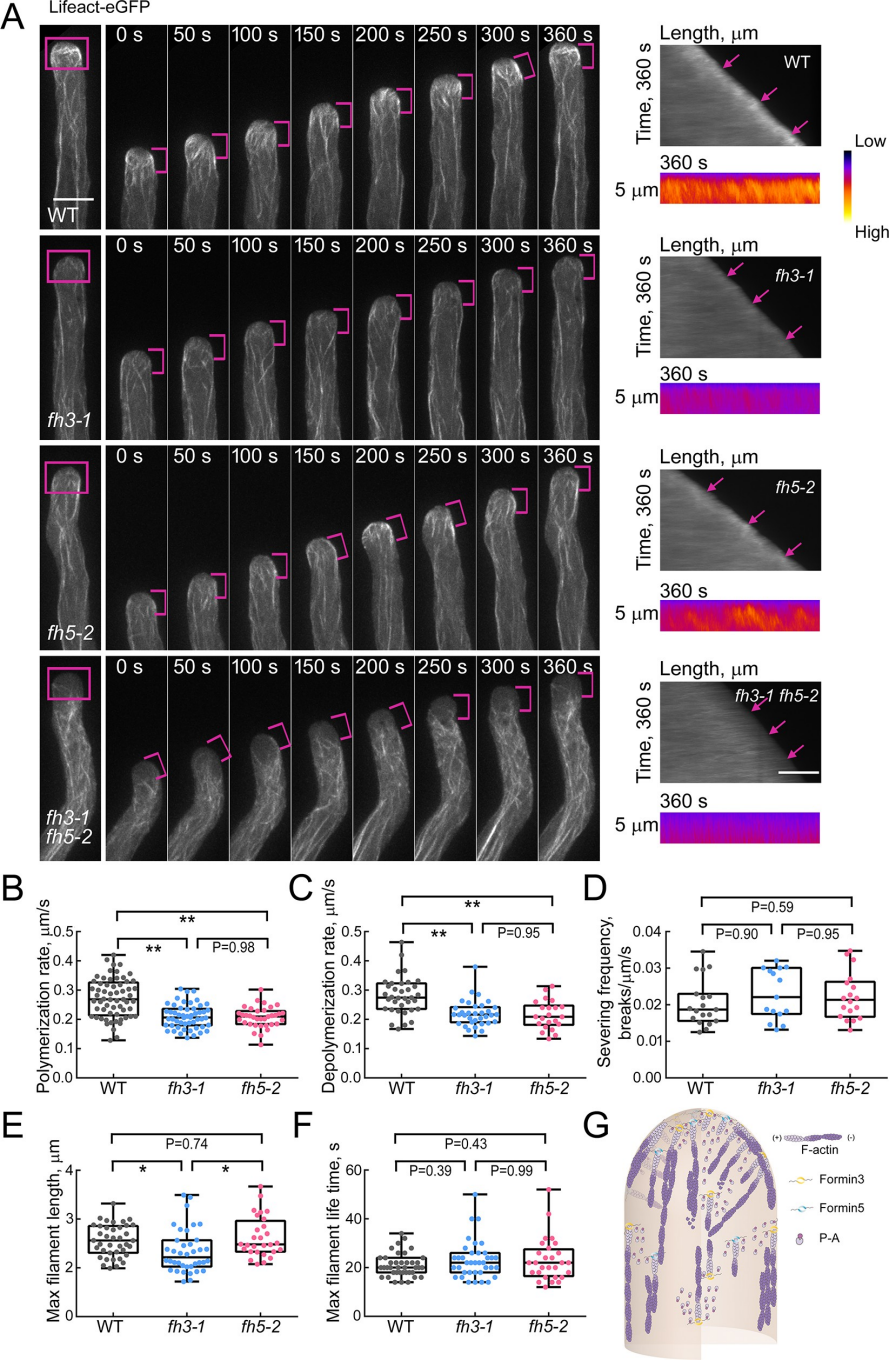
Apical actin polymerization is impaired in formin loss-of-function mutant pollen tubes. (**A**) Time-lapse images of actin filaments in growing WT and *fh3-1*, *fh5-2* and *fh3-1 fh5-2* mutant pollen tubes. The left panels (labeled 0–360 s) show the time-lapse Z-series images of actin filaments decorated with Lifeact-eGFP within pollen tubes of WT and *fh3-1*, *fh5-2* and *fh3-1 fh5-2* mutants. See the entire series in S7 Movie (WT), S8 Movie (*fh3-1*), S9 Movie (*fh5-2*) and S10 Movie (*fh3-1 fh5-2*). The magenta lines indicate the edge of the pollen tubes within the apical and subapical regions. The far left images show the whole pollen tubes; magenta boxes indicate the actin filaments within the apical region. The right upper panels are kymograph analyses of apical actin filaments during pollen tube growth, which show that actin filaments grow out from the apical membrane. Actin filaments originating from the apical membrane are indicated by the magenta arrows. The lower panels show enlarged kymograph images of the pollen tube tip in the top panels after pseudo-color processing. Warm and cold colors indicate high and low fluorescence intensity, respectively. Bar = 10 μm. (**B-F**) Quantification of several parameters associated with actin filament dynamics in the apical region of WT and *fh3-1* and *fh5-2* mutant pollen tubes. *fh3-1 fh5-2* mutants were omitted because it is hard to select individual actin filaments in the apical region of *fh3-1 fh5-2* pollen tubes. (**B**) Quantification of polymerization rate; (**C**) Quantification of depolymerization rate; (**D**) Quantification of actin filament severing frequency; (**E**) Quantification of maximal actin filament length; (**F**) Quantification of maximal filament lifetime. Box plots display the first and third quartiles, split by the median; whiskers extend to include the maximum and minimum values. Data are presented as mean ± SE, statistical comparisons were performed using ANOVA Post-Tukey, *P < 0.05 and **P < 0.01. More than fifteen pollen tubes were measured for each genotype. **(G)** Schematic model describing the function of Formins in nucleating apical actin polymerization in the pollen tube. Based on the facts that (1) AtFH3 and AtFH5 mainly localize to the PM and the endomembrane system as shown in Fig 4, (2) actin polymerization is mainly initiated from the apical membrane, and (3) loss of function of *AtFH3* and/or *AtFH5* impairs apical actin polymerization from the PM (Fig 5A), we propose that the PM-anchored AtFH3 and AtFH5 drive actin polymerization from the membrane by utilizing profilin-actin (P-A) complexes in the cytoplasm within the apical and subapical regions of the pollen tube.

To understand the defective actin filament organization in formin loss-of-function mutant pollen tubes, we traced the dynamics of individual actin filaments decorated with Lifeact-eGFP as described previously [16, 49]. We found that actin filaments are continuously polymerized from the apical membrane during WT pollen tube growth, and consequently form a bright apical actin structure (Fig 5A) [15]. However, we found that apical actin polymerization was impaired in formin loss-of-function mutant pollen tubes, and this affected the formation of the apical actin structure (Figs 5A and S7 and S7–S10 Movies). We next traced the dynamics of individual actin filaments and determined the parameters associated with them. Given that apical actin polymerization is severely impaired in *fh3-1 fh5-2* mutant pollen tubes and it is hard to select individual apical membrane-originated actin filaments for measurement, we only traced the dynamics of individual actin filaments in WT, *fh3-1* and *fh5-2* pollen tubes and carefully compared their dynamic parameters. We found that the elongation rate of actin filaments originating from the apical membrane is reduced significantly in *fh3-1* and *fh5-2* pollen tubes when compared to WT pollen tubes (Fig 5B). This explains to some extent the impairment in the formation of the apical actin structure. In addition, we found that although there is no overt difference in the severing frequency of actin filaments between *fh3-1* and *fh5-2* pollen tubes and WT pollen tubes (Fig 5D), the depolymerization rate of actin filaments is reduced significantly in *fh3-1* and *fh5-2* pollen tubes when compared to WT pollen tubes (Fig 5C). Furthermore, no overt difference was detected in the maximal filament lifetime of apical actin filaments in *fh3-1* and *fh5-2* pollen tubes (Fig 5F), but the maximal filament length of apical actin filaments is reduced significantly in *fh3-1* pollen tubes (Fig 5E), which is very likely due to the reduction in the filament elongation rate of apical actin filaments in *fh3-1* pollen tubes. Given that AtFH3 and AtFH5 are *bona fide* actin nucleation-promoting factors [25, 31], the reduction in the filament depolymerization rate in formin mutant pollen tubes is very likely indirect, and is presumably related to the reduction in the actin filament level in formin mutant pollen tubes. Together, these data suggest that the impairment in the formation of the apical actin structure is mainly caused by the defects in formin-mediated actin filament elongation.

## Discussion

Here we provide direct genetic and cytological evidence showing that two class I formins, AtFH3 and AtFH5, are involved in the regulation of actin polymerization and construction of the apical actin structure in the pollen tube. This finding provides another piece of evidence to show that class I formins are major actin nucleation factors that control apical actin polymerization [4], and further supports the notion that actin assembly mediated by formin/profilin modules is one of the major actin polymerization pathways in the pollen tube [4, 44]. Our study thus substantially enhances our understanding of the molecular mechanisms underpinning rapid actin assembly at pollen tube tips and provides significant insights into actin-mediated regulation of pollen tube growth.

### The class I formins AtFH3 and AtFH5 regulate apical actin polymerization in pollen tubes

We found that AtFH3 and AtFH5 are involved in the regulation of actin polymerization in pollen cells (Figs 2 and 5 and S1, S2 and S7). Within pollen tubes, the reduction in the level of filamentous actin resulting from loss of function of *AtFH3* and *AtFH5* is comparatively more severe at the tip (Figs 2 and 5 and S1 and S7), and this is very likely because the activity of formin is strictly required at the pollen tube tip where active actin polymerization occurs [15]. Correspondingly, we found that formin is relatively concentrated on the PM at the tip of the pollen tube (Fig 4). In support of the role of formin in regulating apical actin polymerization, a recent study showed that a class I formin, LiFH1, is involved in the construction of the actin fringe in the pollen tube [43]. However, LiFH1 is biochemically distinct from AtFH3 and AtFH5 since it has actin filament-bundling activity [43]. If there is a formin that behaves like LIFH1 in *Arabidopsis* pollen tubes, it will be interesting to explore if and how it coordinates with AtFH3 and AtFH5 to regulate apical actin polymerization. In terms of the effect of loss of function of *AtFH3* on the organization of actin filaments in the shank of pollen tubes, our results differ slightly from a previous report that RNAi-mediated downregulation of *AtFH3* causes severe defects including disorganized shank-localized actin cables and depolarized pollen tube growth [31], as we noticed the disorganization of actin filaments in the shank of *fh3* pollen tubes and inhibition of pollen tube growth but not that severe (Figs 1, 2 and 5). We do not currently know the reason for this, but it could be due to off-target effects derived from the RNAi-mediated downregulation approach. For instance, this RNAi construct might target to other pollen-expressed formins. If this is indeed the case, it explains why loss of function of *AtFH3* and/or *AtFH5* causes weak phenotype in term of pollen tube growth. Certainly, we also cannot rule out the possibility that Arp2/3 complex might take a partial role in nucleating actin assembly to compensate for the loss of AtFH3 and/or AtFH5 in pollen tubes, although Arp2/3 complex and formins nucleate actin assembly using different biochemical mechanisms.

Nonetheless, we convincingly demonstrate that AtFH3 and AtFH5 contribute to actin polymerization at the pollen tube tip and AtFH3 plays a more dominant role than AtFH5 in this process (Figs 2 and 5 and S1 and S7). This is actually consistent with the fact that the expression of *AtFH3* is more abundant than *AtFH5* in pollen (https://www.genevestigator.com/gv/index.jsp). Considering these results along with observations showing that actin filaments are mainly generated from the apical membrane (Figs 5A and S7) [15, 16, 44, 50, 51], it is fair for us to propose that the membrane-anchored class I formins, AtFH3 and AtFH5, drive actin polymerization by utilizing profilin-actin complexes in the cytoplasm within the apical region of the pollen tube (Fig 5G). We showed that AtFH3 and AtFH5 are abundant within the cytoplasm of the growth domain and are presumably localized on vesicles (Fig 4B and 4E). These observations suggest that the activity of formins on the surface of vesicles is maintained at a very low level since no obvious actin polymerization was detected from the surface of vesicles in pollen tubes. Furthermore, it was reported that apical actin polymerization occurs concurrently with and is required for pollen tube growth [15]. The mechanism that activates formins on the PM during pollen tube growth is of great interest. Compared to non-plant formins, plant formins lack the GTPase-binding domain (GBD) and the diaphanous autoregulatory domain (DAD) that are crucial for the regulation of their actin nucleation activity [18, 19, 24]. The molecular mechanisms that tightly regulate the activity of plant formins remain a mystery.

### Functional specification of AtFH3 and AtFH5 in the pollen tube

We found that AtFH3 and AtFH5 both localize to the PM, and exhibit distinct PM localization patterns (Fig 4). Considering that the intracellular localization of AtFH3 and AtFH5 is determined by their TM domains (S6 Fig), the distinct PM localization pattern of AtFH3 and AtFH5 suggests that their TM domains have distinct functions. In support of this notion, we found that substitution of the TM domain of AtFH3 with that of AtFH5 enables AtFH3 to exhibit a PM localization pattern similar to that of AtFH5 (S6B Fig). Our data suggest that, although AtFH3 and AtFH5 belong to the same subclass, functional divergence of their TM domains has endowed them with distinct PM localization patterns. The two proteins might consequently perform distinct roles in regulating membrane-originated actin polymerization within the pollen tube. The function of formins, as regulators of actin dynamics, is achieved through their FH1FH2 domain [18]. It remains to be determined whether the C-terminal FH1FH2 domain of AtFH3 and AtFH5 might have distinct actin regulatory functions. Previous studies revealed that both AtFH3 and AtFH5 are *bona fide* actin nucleation factors [25, 31], but no side-by-side comparison has been performed. Given that actin monomers were predicted to be buffered by an equal amount of profilin in pollen [9], AtFH3 and AtFH5 might differ in their ability to utilize profilin-actin complexes in the pollen tube. There are at least five actin isovariants and two profilin isovariants in *Arabidopsis* pollen, and they were reported to be distributed uniformly in the cytoplasm of the pollen tube overall [44, 52, 53]. It is possible that they may form different profilin-actin complexes within the cytoplasm. In this regard, AtFH3 and AtFH5 might have preference for certain profilin-actin complexes in the pollen tube. Consequently, the combination of different actin, profilin and formin isovariants may fine-tune the actin polymerization machinery to meet the demands of rapid pollen tube growth. In support of this speculation, a previous report showed that AtFH4 interacts specifically with profilin 2 (PFN2) rather than PFN3 [41].

## Materials and methods

### Plant materials and growth conditions

The *Arabidopsis* plants were cultured at 22°C under a 16-h light/8-h dark cycle. The T-DNA insertion mutants, *fh3-1* (Salk_150350), *fh5-2* (Salk_044464), and *fh5-3* (Salk_152090) were obtained from Nottingham *Arabidopsis* Stock Center on the Columbia-0 ecotype (Col-0) background. They were backcrossed with Col-0 three times before the subsequent phenotypic analyses. *fh3-2* (CSHL_GT24923) was obtained from Cold Spring Harbor Laboratory and backcrossed with Col-0 three times before the phenotypic characterization. The genotyping of *fh3-1*, *fh5-2* and *fh5-3* was performed using primer pairs *fh3-1* salk_150350-LP/*fh3-1* salk_150350-RP and *fh5-2* salk_044464-LP/*fh5-2* salk_044464-RP, and *fh5-3* salk_152090-LP/*fh5-3* salk_152090-RP (S1 Table), respectively, in combination with Salk_LB 1.3 (S1 Table). The genotyping of *fh3-2* was performed using primer pair *fh3-2* CSHL_GT24923-LP/*fh3-2* CSHL_GT24923-RP along with Ds3-1 (see S1 Table). The T-DNA insertion mutant *fh5-2* has been characterized previously [25]. To determine the functional coordination between *AtFH3* and *AtFH5*, *fh3-1 fh5-2* and *fh3-2 fh5-3* double mutants were generated by crossing *fh3-1* with *fh5-2* or *fh3-2* with *fh5-3*.

### Complementation and determination of the intracellular localization of AtFH3 and AtFH5

To complement *fh3-1* and *fh5-2* and indicate the intracellular localization of AtFH3 and AtFH5, GFP fusion constructs of *AtFH3* and *AtFH5* driven by their own promoters were generated. To generate the *AtFH3-eGFP* fusion construct, the nucleotide sequence containing the promoter and genomic region of *AtFH3* were amplified from *Arabidopsis* genomic DNA with the primer pair *AtFH3*pg-*Pst*I-F/*AtFH3*pg-*Kpn*I-R (see S1 Table) and *eGFP* was amplified from pCAMBIA1301 carrying eGFP with eGFP-*Sac*I-F/eGFP-*Eco*RI-R (see S1 Table). The PCR products were subsequently moved into pCAMBIA1301 to generate pCAMBIA1301-gFormin3-eGFP. To generate the *AtFH5-eGFP* fusion construct, the promoter sequence and the genomic sequence of *AtFH5* were amplified with the primer pairs *AtFH5*pro-F/*AtFH5*pro-R and *AtFH5*genomic-F/*AtFH5*genomic-R (see S1 Table), respectively. Given that no suitable restriction enzyme sites were available, the *AtFH5* genomic sequence was mutated to disrupt an internal *Sac*I restriction site so that *Sac*I could then be used for the subsequent cloning. The *AtFH5* genomic sequence was amplified with primers g*AtFH5*-Mut-F/g*AtFH5*-Mut-R using the *AtFH5* genomic sequence as the template. The product was subsequently moved into *pCAMBIA1301* to generate the final *pCAMBIA1301-gFormin5-eGFP* construct. The constructs *gFormin3-eGFP-pCAMBIA1301* and *gFormin5-eGFP-pCAMBIA1301* were transformed into *fh3-1* and *fh5-2* to generate the transgenic plants, *gFormin3-eGFP-pCAMBIA1301*;*fh3-1* and *gFormin5-eGFP-pCAMBIA1301*;*fh5-2*, respectively, using the agro bacteria-mediated flower-dipping method [54]. The transgenic plants at T3 were used for the subsequent analysis. To determine whether the intracellular localization pattern of AtFH3 is determined by its N-terminus, which contains signal peptide (SP) and transmembrane (TM) domain, we amplified the sequence containing both SP and TM of AtFH3 (AtFH3-SP-TM) using primer pair AtFH3-SPTM-F/AtFH3-SPTM-R (S1 Table). The PCR product of AtFH3-SP-TM, along with the Lat52 promoter amplified with pair Lat52-F/Lat52-R (S1 Table), was moved into pCAMBIA1301 to generate pCAMBIA1301-Lat52pro-AtFH3-SP-TM-eGFP. The construct was subsequently transformed into WT *Arabidopsis* plants. Pollen derived from the transgenic plants was germinated on solid GM for 2 h, then observed under an Olympus FV1000MPE multiphoton laser scanning confocal microscope equipped with a 100× objective (numerical aperture of 1.4). Samples were excited under a 488-nm argon laser with the emission wavelength set at 505–605 nm.

### Domain swapping

To replace the TM domain of AtFH3 with that of AtFH5, overlap PCR was performed to amplify the sequence of the promoter of *AtFH3* (AtFH3pro) and AtFH5-SP-TM with primer pairs 3+5TM F2/3+5TM R2 and 3+5TM F3/3+5TM R3 (S1 Table) using AtFH3pro and AtFH5-SP-TM as the template, respectively. Subsequently, the overlap products were amplified specifically with primer pair 3+5TM F1-*Xba*I/3+5TM R1-*Kpn*I (S1 Table). Given that no suitable restriction sites were available, the AtFH3pro-AtFH5-SP-TM genomic sequence was subsequently mutated using PCR with primer pair 3+5TM-Mut-F/3+5TM-Mut-R (S1 Table) to disrupt an internal *Pst*I restriction site in order to facilitate subsequent cloning. The sequences of AtFH3pro-AtFH5-SP-TM and AtFH3 FH1FH2 were then amplified with primer pairs 3+5TM F2-*Pst*I/3+5TM R2-*Xba*I and AtFH3 FH1FH2-F/AtFH3 FH1FH2-R (S1 Table), respectively. The error-free PCR products were subsequently moved into pCAMBIA1301 to generate pCAMBIA1301-AtFH3pro-AtFH5-SP-TM-AtFH3-FH1FH2-eGFP. The construct pCAMBIA1301-AtFH3pro-AtFH5-SP-TM-AtFH3-FH1FH2-eGFP was finally transformed into *fh3-1* to generate the transgenic plants, pCAMBIA1301-AtFH3pro-AtFH5-SP-TM-AtFH3-FH1FH2-eGFP;*fh3-1*. The transgenic *Arabidopsis* plants at T3 were used for the subsequent analysis.

### Quantitative (q)RT-PCR

qRT-PCR was performed to determine the transcript levels of *AtFH3* and/or *AtFH5* in the formin T-DNA insertion mutants. Total RNA was isolated from pollen derived from WT (wild-type), *fh3-1*, *fh3-2*, *fh5-2*, *fh5-3*, *fh3-1 fh5-2* and *fh3-2 fh5-3* plants using TRIzol reagent (Invitrogen) according to the manufacturer’s instructions, and cDNA was subsequently synthesized using MMLV reverse transcriptase (Promega) with oligo-d(T)_18_. To determine the *AtFH3* transcript levels, partial coding region sequences of *AtFH3* were amplified with primer pairs *AtFH3*-F1/*AtFH3*-R1 and *AtFH3*-F2/*AtFH3*-R2 (see S1 Table). To determine the *AtFH5* transcript levels, the partial coding region of *AFH5* was amplified with the primer pair *AtFH5*-F/*AtFH5*-R. To determine the *AtFH3* and *AtFH5* transcript levels in the complementation plants, the primer pairs *AtFH3*-F2/*AtFH3*-R2 and *AtFH5*-F/*AtFH5*-R (see S1 Table) were used, respectively. The internal control was *eIF4A*, which was amplified with the primer pair q-*eIF4A*-F/q-*eIF4A*-R (see S1 Table). The real-time PCR data were analyzed with the method of Livak (2^-ΔΔCt^) [55].

### Pollen germination and tube growth measurements

*In vitro Arabidopsis* pollen germination was performed according to previously described methods [56]. Briefly, pollen was isolated from newly opened flowers and placed on pollen germination medium [GM: 1 mM CaCl_2_, 1 mM Ca(NO_3_)_2_, 1 mM MgSO_4_, 0.01% (w/v) H_3_BO_3_, and 18% (w/v) sucrose solidified with 0.8% (w/v) agar, pH 6.9~7.0]. The plates were cultured at 28°C under moist conditions. After 2 h of culture, the pollen germination rate was quantified by observing pollen grains and pollen tubes under an IX71 microscope (Olympus) equipped with a 10× objective. Images were collected with a Retiga EXi Fast 1394 CCD (charge-coupled device) camera using Image-Pro Express 6.3 software. To calculate the pollen germination percentage, a minimum of 300 pollen grains was counted in each experiment. At least three experiments were performed.

To accurately calculate the pollen tube growth rate, we developed a new method based on calculating the slope of a kymograph of a single growing pollen tube. Briefly, after the pollen tube grew to an average length of approximately 200–300 μm, the solid pollen germination medium containing the germinating pollen was moved to a circular plate (Cat# D35-20-1-N, In Vitro Scientific) under an IX71 microscope (Olympus) equipped with a 4× objective. A microscope field containing at least 15~20 pollen tubes was identified, and the growth of individual pollen tubes was monitored by collecting time-lapse images (about 15–20 images in total) at time intervals of 1 min. A kymograph was created along the growth direction at the center of the growing pollen tube and the growth rate of the pollen tube was calculated from the slope of the kymograph. The experiments were repeated at least three times.

### FM dye staining of living pollen tubes

Pollen tubes were stained with the lipophilic dye FM4-64 (Invitrogen). The loading of pollen tubes with FM4-64 was achieved by direct addition of FM4-64 dye (5 μM in liquid pollen germination medium) on the surface of solid pollen germination medium. After incubation with FM4-64 solution for 15 min, images were captured with an Olympus FV1000MPE multiphoton laser scanning confocal microscope as described above. FM4-64 dye was excited with an argon laser at 546 nm, and the emission wavelength was set in a range of 600–650 nm.

### Actin staining with Alexa-488/568 phalloidin in fixed pollen grains and pollen tubes

To reveal the organization of the actin cytoskeleton in pollen grains and pollen tubes, pollen grains were germinated for 2 h on solid GM, then subjected to fixation and staining with Alexa-488/568 phalloidin as described previously [52, 57]. Actin filaments were observed with an Olympus FV1000MPE multiphoton laser scanning confocal microscope equipped with a 100× objective (numerical aperture of 1.4). The fluorescent phalloidin was excited with an argon laser at 488 nm and 560 nm, and the emission wavelength was set in the range of 505–605 nm and 650–700 nm, respectively. The relative amount of actin filaments in pollen grains and pollen tubes was quantified by measuring the fluorescence pixel intensity using ImageJ software (http://rsbweb.nih.gov/ij/; version 1.46). At least three experiments were performed. The organization of apical actin filaments or bundles was quantified by determining the angles formed between each apical actin filament or bundle and the growth axis of pollen tubes, which was performed with ImageJ roughly as described previously for the quantification of the angles formed between longitudinal actin cables and the growth axis of the pollen tube in the shank region [56]. To ensure that each apical actin filament or bundle was analyzed only once, three to four optical sections were excluded for analysis in each pollen tube. Since we do not know the polarity of apical actin filaments or bundles, we only selected the small angles. More than 200 apical actin filaments or bundles from 10 pollen tubes were measured for each genotype.

### Visualization and quantification of actin filament dynamics in living pollen tubes

In order to visualize the dynamics of actin filaments in pollen tubes, the actin marker Lifeact-eGFP was introduced into formin loss-of-function mutants (*fh3-1*, *fh3-2*, *fh5-2*, *fh5-3*, *fh3-1 fh5-2* and *fh3-2 fh5-3*) by crossing the mutants with transgenic WT plants harboring *Lat52*:*Lifeact-eGFP* [16]. Time-lapse Z-series images were collected every 2 s using MetaMorph software with the step size set at 0.7 μm. The dynamics of individual actin filaments were quantified by measuring dynamic parameters, including the elongation rate, depolymerization rate, severing frequency, maximum filament length and maximum filament lifetime as described previously [16, 57]. At least ten pollen tubes for each genotype were analyzed. A kymograph taken along the growth direction at the center of the pollen tube was created to analyze the F-actin intensity along the growing pollen tube as described previously [16].

### Visualization and quantification of RabA4b-positive vesicles

To observe the tip-directed vesicle transport in pollen tubes, YFP-RabA4b was introduced into the formin loss-of-function mutants by crossing the mutants with transgenic WT plants expressing *Lat52*:*YFP-RabA4b* [48, 58]. Pollen from the resulting plants was germinated on solid GM at 28°C, and when the pollen tubes reached about 150 μm, they were imaged under an Olympus FV1000MPE multiphoton laser scanning confocal microscope equipped with a 100× objective (numerical aperture of 1.4). Samples were excited under a 488-nm laser with the emission wavelength set at 505–605 nm. Optical sections were scanned with the step size set at 0.7 μm. For the fluorescence recovery after photobleaching (FRAP) experiments, apical regions were bleached for 3 s using a 488-nm laser at 100% power and a 405-nm laser at 45% power. Fluorescence recovery was recorded at 2 s intervals for 200s with a 488-nm laser at 10% power. To determine the recovery rate, the mean gray value of the apical region (0–5 μm away from the tip) was measured using ImageJ software and plotted against the elapsed time as described previously [52, 58]. Experiments were repeated at last 20 times and the values of YFP-RabA4b fluorescence were averaged and used for subsequent exponential curve fitting as described previously [52].

## Supporting information

S1 Fig Loss of function of *AtFH3* and *AtFH5* reduces the amount of apical actin filaments in pollen tubes. **(A)** Micrographs of pollen tubes stained with Alexa-488 phalloidin. The right panel shows the 2D distribution of fluorescence pixel intensity of actin filaments within the apical region. In the left panel, white arrows indicate some short and fragmented actin bundles in *fh3-2 fh5-3* pollen tubes within the red boxed region. Bar = 10 μm. **(B)** Quantification of the relative fluorescence intensity of actin filaments within the red boxed apical and subapical region of pollen tubes shown in (**A**). Data are presented as mean ± SE, statistical comparisons were performed using ANOVA Post-Tukey, * P < 0.05, ** P < 0.01. (**C**) Quantification of the fluorescence intensity of actin filaments within the shank regions of pollen tubes. Data were presented as mean ± SE, statistical comparisons were performed using ANOVA Post-Tukey, *P < 0.05, and **P < 0.01. (**D**) Plot of the average degrees of angles formed between actin filaments and the pollen tube growth axis within the shank regions of pollen tubes. The way of the measurement of angles see the description in the legend of Fig 2F. Data represent mean ± SE. More than 150 actin filaments were measured from 10 pollen tubes for each genotype. Statistical comparisons were performed using ANOVA Post-Tukey, **P < 0.01.
(TIF)

S2 Fig Loss of function of *AtFH3* and *AtFH5* reduces the amount of actin filaments in pollen grains. (**A**) Micrographs of pollen grains stained with Alexa-488 phalloidin. For each genotype, the upper panel shows the Z-projection image. The lower panels are the optical sections of the stained pollen grains. Bars = 10 μm. (**B**) Quantification of the average fluorescence intensity of pollen grains. Data are presented as mean ± SD, statistical comparisons were performed using ANOVA Post-Tukey, **P < 0.01.
(TIF)

S3 Fig AtFH3-eGFP and AtFH5-eGFP are fully functional. (**A**) Quantitative RT-PCR analysis shows the transcript level of *AtFH3* in pgAtFH3. *AtFH3* expression in the *fh3-1* complemented line #5 was restored to that of WT. *eIF4A* was used as an internal control. (**B**) Images of pollen tubes derived from WT, *fh3-1* and the restored line (#5) after staining with Alexa-568 phalloidin are presented. The dashed blue lines indicate the base of the subapical region that was used to quantify the fluorescence intensity of actin filaments. Bar = 10 μm. (**C**) Determination of the fluorescence intensity of the actin filaments in the apical region of pollen tubes. Date are presentend as mean ± SE, statistical comparisons were performed using ANOVA Post-Tukey, **P < 0.01. (**D**) Plot of the average degrees of angles formed between actin filaments and the pollen tube growth axis within the apical region. The way of the measurement of angles between actin filaments and pollen tube growth axis see the description in legend of Fig 2F. Data represent mean ± SE. More than 150 actin filaments were measured from 10 pollen tubes for each genotype. Statistical comparisons were performed using ANOVA Post-Tukey, **P < 0.01. (**E**) Quantitative RT-PCR analysis shows the transcript level of *AtFH5* in pgAtFH5. *AtFH5* expression in the *fh5-2* complemented line #1 was restored to that of WT. *eIF4A* was used as an internal control. (**F**) Images of pollen tubes derived from WT, *fh5-2* and the restored line (#1) after staining with Alexa-568 phalloidin are presented. The dashed blue lines indicate the base of the subapical region that was used to quantify the fluorescence intensity of actin filaments. Bar = 10 μm. (**G**) Determination of the fluorescence intensity of actin filaments in the apical region of pollen tubes. Date are presentend as mean ± SE, statistical comparisons were performed using ANOVA Post-Tukey, **P < 0.01.
(TIF)

S4 Fig Intracellular localization of AtFH3-eGFP and AtFH5-eGFP in pollen tubes after plasmolysis. Pollen tubes derived from *AtFH3pro*:*AtFH3-eGFP;fh3-1* and *AtFH5pro*:*AtFH5-eGFP;fh5-2* plants were subjected to treatment with 15% mannitol in germination medium. Pollen tubes were observed by confocal micropy after treatment for 3 min. Bars = 10 μm.
(TIF)

S5 Fig AtFH3 and AtFH5 are still able to target to the PM of pollen tubes after treatment with 100 nM Latrunculin B. To determine whether the disruption of actin filaments affects PM targeting of AtFH3 and AtFH5, 100 nM latrunculin B (LatB) was applied onto the surface of solid pollen germination medium containing pollen for 30 min. Actin was then stained with Alexa488-phalloidin and pollen tubes were directly visualized with confocal microscopy. (**A**) Actin filaments stained with Alexa488-phalloidin in WT pollen tubes. Actin filaments are obviously depolymerized in WT pollen tubes after treatment with 100 nM LatB for 30 min. The projection image is presented and yellow asterisks indicate the disrupted actin filaments. Bar = 10 μm. (**B**) Distribution of AtFH3-eGFP in pollen tubes derived from *AtFH3pro*:*FH3-eGFP;fh3-1* plants treated with 100 nM LatB. FM4-64 staining was employed to reveal the PM and endocytic vesicles. The magenta asterisks indicate the localization of AtFH3-eGFP on the PM. Bar = 10 μm. (**C**) Distribution of AtFH5-eGFP in pollen tubes derived from *AtFH5pro*:*FH5-eGFP;fh5-2* plants treated with 100 nM LatB. FM4-64 staining was employed to reveal the PM and endocytic vesicles. The magenta asterisks indicate the localization of AtFH5-eGFP on the PM. Bar = 10 μm.
(TIF)

S6 Fig The localization of AtFH3 is determined by its N-terminal SP-TM domain. (**A**) Distribution of AtFH3-SP-TM-eGFP protein in ungerminated and germinated pollen derived from *Arabidopsis* plants harboring pCAMBIA1301-Lat52pro-AtFH3-SP-TM-eGFP. Medial optical sections are presented. The magenta asterisks indicate the localization of AtFH3-SP-TM-eGFP on the PM. Co-localization of AtFH3-SP-TM protein with FM4-64-stained plasma membrane and endocytic vesicles is presented in the right panel. Bars in left and right panels are 5 μm and 10 μm, respectively. (**B**) Distribution of AtFH5-SP-TM-AtFH3-FH1FH2-eGFP protein in ungerminated and germinated pollen derived from AtFH3pro-AtFH5-SP-TM-AtFH3-FH1FH2-eGFP;*fh3-1* plants. Medial optical sections are presented. The magenta asterisks indicate the localization of the fusion protein on the PM. Co-localization of AtFH5-SP-TM-AtFH3-FH1FH2-eGFP protein with FM4-64-stained plasma membrane and endocytic vesicles is presented in the right panel. Bars in left and right panels are 5 μm and 10 μm, respectively.
(TIF)

S7 Fig The amount of F-actin is reduced within the apical region of *fh3*, *fh5*, and *fh3 fh5* pollen tubes. Time-lapse images of actin filaments revealed by decoration with Lifeact-eGFP in growing WT and *fh3-2*, *fh5-3* and *fh3-2 fh5-3* mutant pollen tubes. Red boxes indicate the apical region in pollen tubes. The right panels are kymograph images of the growing pollen tube tips after pseudo-color processing. Warm and cold colors indicate high and low fluorescence intensity, respectively. Bar = 10 μm.
(TIF)

S1 Movie Movement of RabA4b-positive vesicles in a WT pollen tube. Movie corresponding to the time-lapse image series of the WT pollen tube shown in Fig 3D. Images were captured every 2 s and are displayed at 10 frames per second.
(AVI)

S2 Movie Movement of RabA4b-positive vesicles in a *fh3-1* pollen tube. Movie corresponding to the time-lapse image series of the *fh3-1* pollen tube shown in Fig 3D. Images were captured every 2 s and are displayed at 10 frames per second.
(AVI)

S3 Movie Movement of RabA4b-positive vesicles in a *fh5-2* pollen tube. Movie corresponding to the time-lapse image series of the *fh5-2* pollen tube shown in Fig 3D. Images were captured every 2 s and are displayed at 10 frames per second.
(AVI)

S4 Movie Movement of RabA4b-positive vesicles in a *fh3-1 fh5-2* pollen tube. Movie corresponding to the time-lapse image series of the *fh3-1 fh5-2* pollen tube shown in Fig 3D. Images were captured every 2 s and are displayed at 10 frames per second.
(AVI)

S5 Movie Intracellular localization and dynamics of AtFH3-eGFP in a *AtFH3pro*:*FH3-eGFP;fh3-1* pollen tube. Movie corresponding to the time-lapse image series shown in Fig 4B. Images were captured every 3 s and are displayed at 30 frames per second.
(AVI)

S6 Movie Intracellular localization and dynamics of AtFH5-eGFP in a *AtFH5pro*:*FH5-eGFP;fh5-2* pollen tube. Movie corresponding to the time-lapse image series shown in Fig 4E. Images were captured every 3 s and are displayed at 30 frames per second.
(AVI)

S7 Movie Dynamics of actin filaments in a WT pollen tube. Movie corresponding to the time-lapse image series shown in Fig 5A. Images were captured every 2 s and are displayed at 30 frames per second.
(AVI)

S8 Movie Dynamics of actin filaments in a *fh3-1* pollen tube. Movie corresponding to the time-lapse image series shown in Fig 5A. Images were captured every 2 s and are displayed at 30 frames per second.
(AVI)

S9 Movie Dynamics of actin filaments in a *fh5-2* pollen tube. Movie corresponding to the time-lapse image series shown in Fig 5A. Images were captured every 2 s and are displayed at 30 frames per second.
(AVI)

S10 Movie Dynamics of actin filaments in a *fh3-1 fh5-2* pollen tube. Movie corresponding to the time-lapse image series shown in Fig 5A. Images were captured every 2 s and are displayed at 30 frames per second.
(AVI)

S1 Table Primers used in this study. (DOCX)
