# Supplementary figures and images for: Arabidopsis class I formins control membrane-originated actin polymerization at pollen tube tips
Source: PLoS Genet. 2018 Nov 12;14(11):e1007789. doi: 10.1371/journal.pgen.1007789 (PMC6258422; doi:10.1371/journal.pgen.1007789)

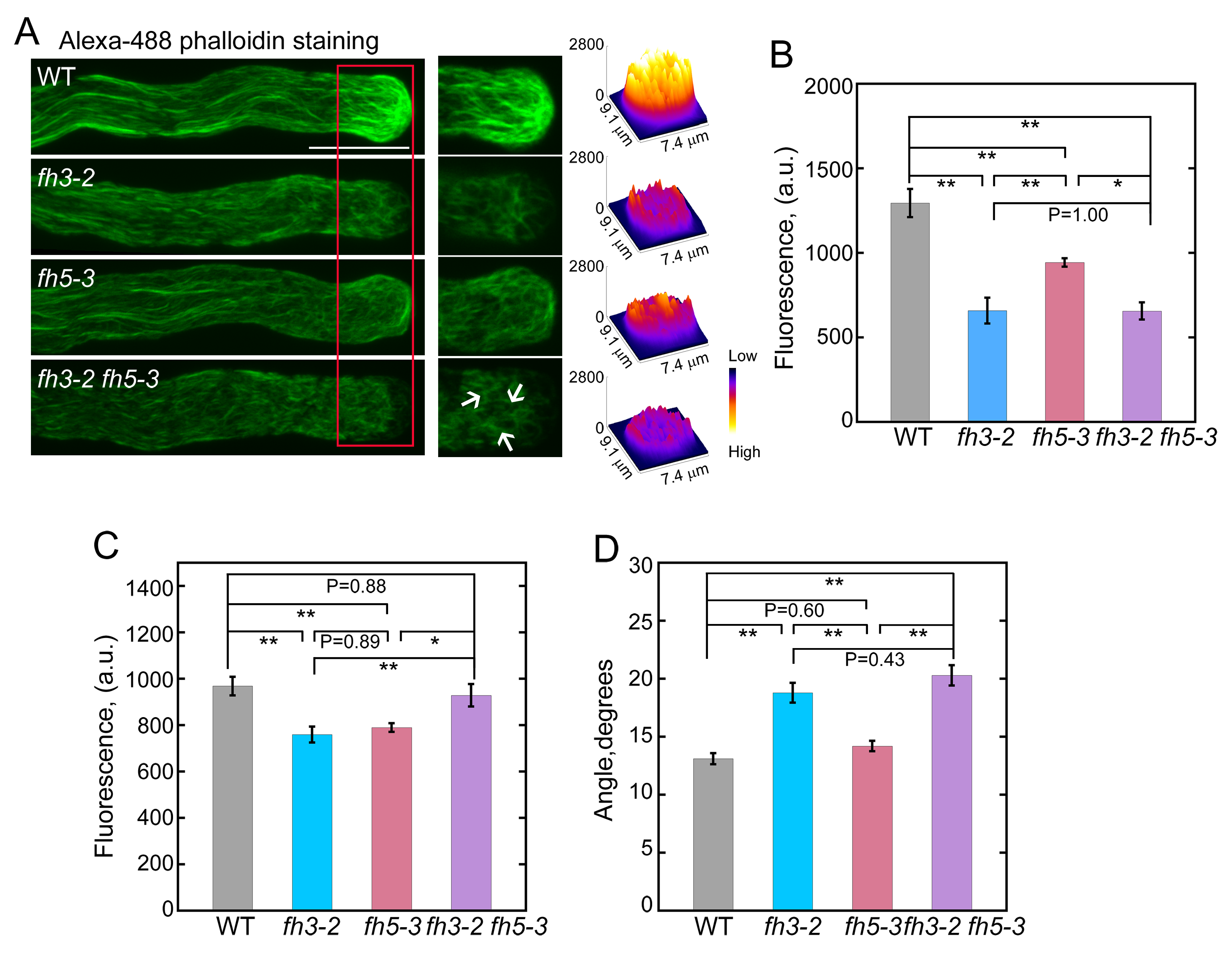

Supplement: S1 Fig — (A) Micrographs of pollen tubes stained with Alexa-488 phalloidin. The right panel shows the 2D distribution of fluorescence pixel intensity of actin filaments within the apical region. In the left panel, white arrows indicate some short and fragmented actin bundles in fh3-2 fh5-3 pollen tubes within the red boxed region. Bar = 10 μm. (B) Quantification of the relative fluorescence intensity of actin filaments within the red boxed apical and subapical region of pollen tubes shown in (A). Data are presented as mean ± SE, statistical comparisons were performed using ANOVA Post-Tukey, * P < 0.05, ** P < 0.01. (C) Quantification of the fluorescence intensity of actin filaments within the shank regions of pollen tubes. Data were presented as mean ± SE, statistical comparisons were performed using ANOVA Post-Tukey, *P < 0.05, and **P < 0.01. (D) Plot of the average degrees of angles formed between actin filaments and the pollen tube growth axis within the shank regions of pollen tubes. The way of the measurement of angles see the description in the legend of Fig 2F. Data represent mean ± SE. More than 150 actin filaments were measured from 10 pollen tubes for each genotype. Statistical comparisons were performed using ANOVA Post-Tukey, **P < 0.01. (TIF) [file pgen.1007789.s001.tif]

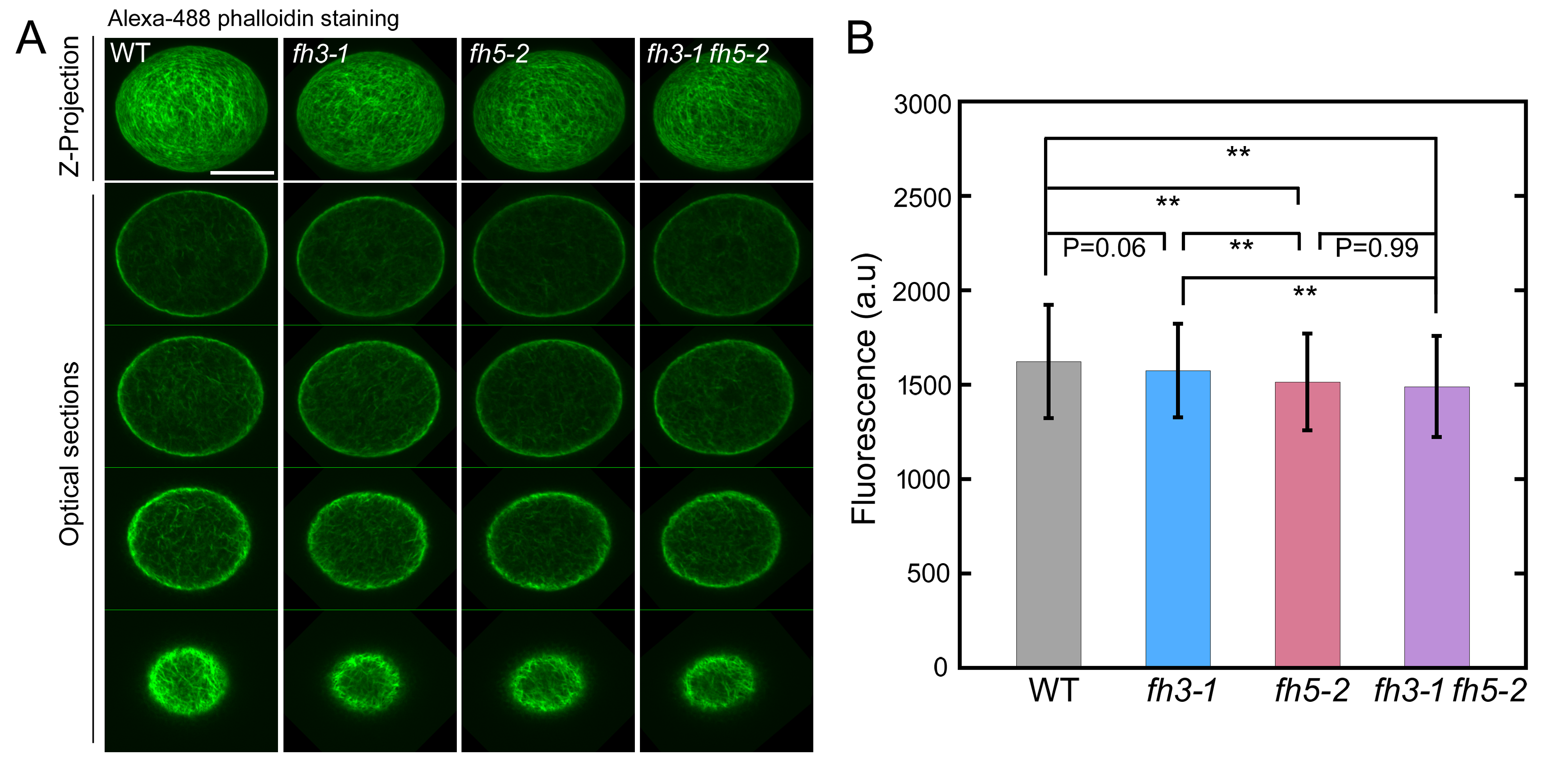

Supplement: S2 Fig — (A) Micrographs of pollen grains stained with Alexa-488 phalloidin. For each genotype, the upper panel shows the Z-projection image. The lower panels are the optical sections of the stained pollen grains. Bars = 10 μm. (B) Quantification of the average fluorescence intensity of pollen grains. Data are presented as mean ± SD, statistical comparisons were performed using ANOVA Post-Tukey, **P < 0.01. (TIF) [file pgen.1007789.s002.tif]

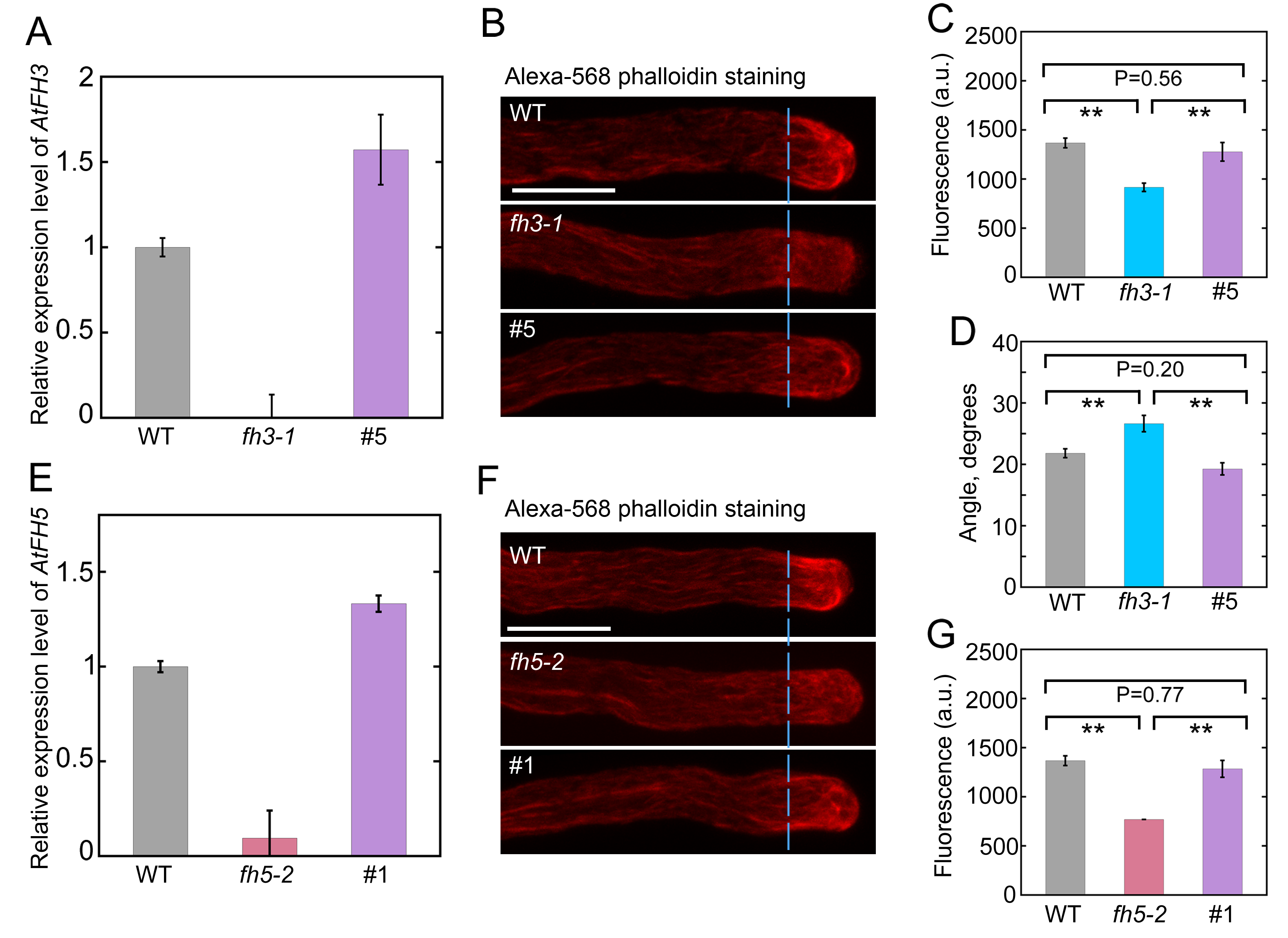

Supplement: S3 Fig — (A) Quantitative RT-PCR analysis shows the transcript level of AtFH3 in pgAtFH3. AtFH3 expression in the fh3-1 complemented line #5 was restored to that of WT. eIF4A was used as an internal control. (B) Images of pollen tubes derived from WT, fh3-1 and the restored line (#5) after staining with Alexa-568 phalloidin are presented. The dashed blue lines indicate the base of the subapical region that was used to quantify the fluorescence intensity of actin filaments. Bar = 10 μm. (C) Determination of the fluorescence intensity of the actin filaments in the apical region of pollen tubes. Date are presentend as mean ± SE, statistical comparisons were performed using ANOVA Post-Tukey, **P < 0.01. (D) Plot of the average degrees of angles formed between actin filaments and the pollen tube growth axis within the apical region. The way of the measurement of angles between actin filaments and pollen tube growth axis see the description in legend of Fig 2F. Data represent mean ± SE. More than 150 actin filaments were measured from 10 pollen tubes for each genotype. Statistical comparisons were performed using ANOVA Post-Tukey, **P < 0.01. (E) Quantitative RT-PCR analysis shows the transcript level of AtFH5 in pgAtFH5. AtFH5 expression in the fh5-2 complemented line #1 was restored to that of WT. eIF4A was used as an internal control. (F) Images of pollen tubes derived from WT, fh5-2 and the restored line (#1) after staining with Alexa-568 phalloidin are presented. The dashed blue lines indicate the base of the subapical region that was used to quantify the fluorescence intensity of actin filaments. Bar = 10 μm. (G) Determination of the fluorescence intensity of actin filaments in the apical region of pollen tubes. Date are presentend as mean ± SE, statistical comparisons were performed using ANOVA Post-Tukey, **P < 0.01. (TIF) [file pgen.1007789.s003.tif]

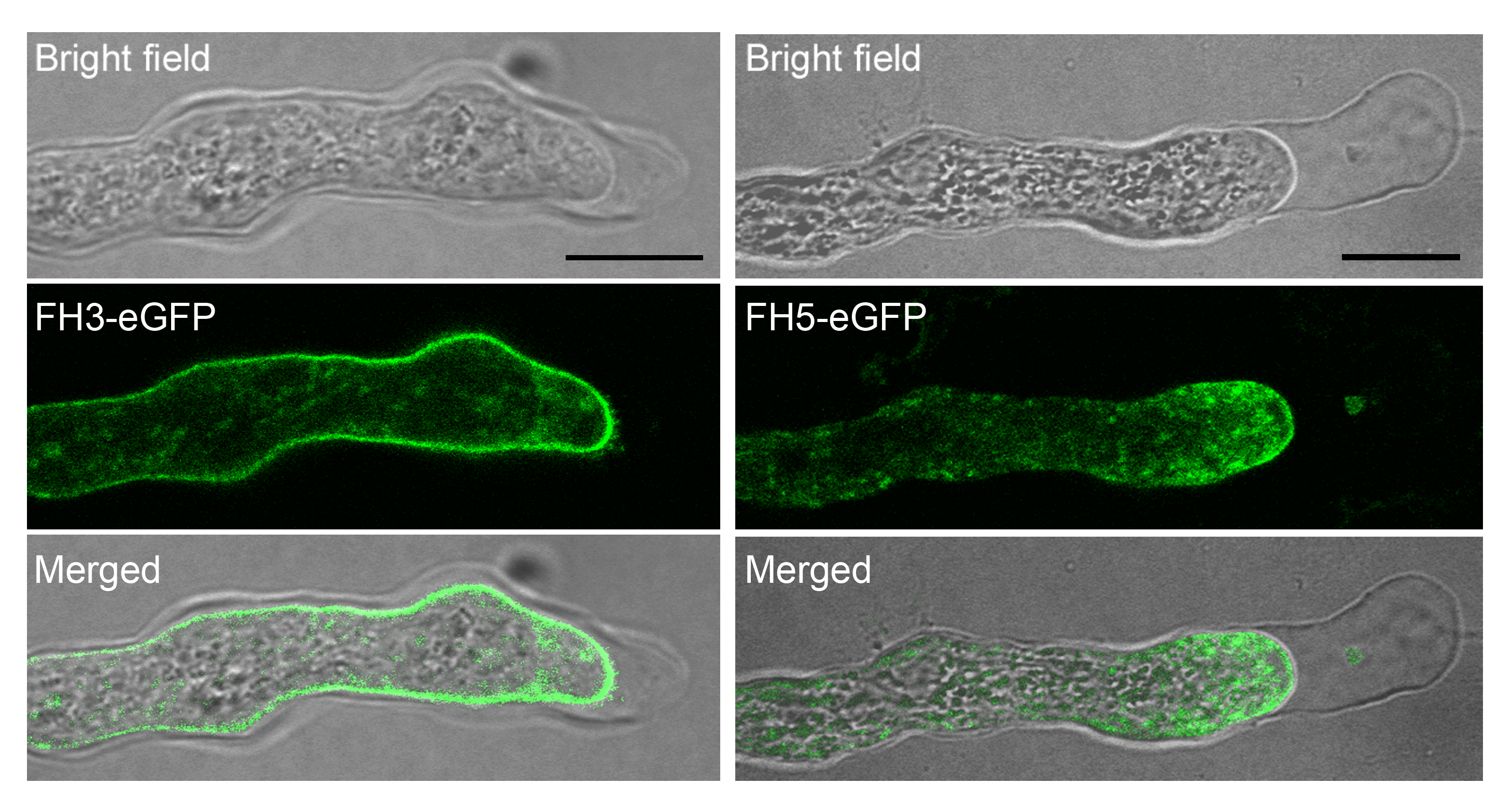

Supplement: S4 Fig — Pollen tubes derived from AtFH3pro:AtFH3-eGFP;fh3-1 and AtFH5pro:AtFH5-eGFP;fh5-2 plants were subjected to treatment with 15% mannitol in germination medium. Pollen tubes were observed by confocal micropy after treatment for 3 min. Bars = 10 μm. (TIF) [file pgen.1007789.s004.tif]

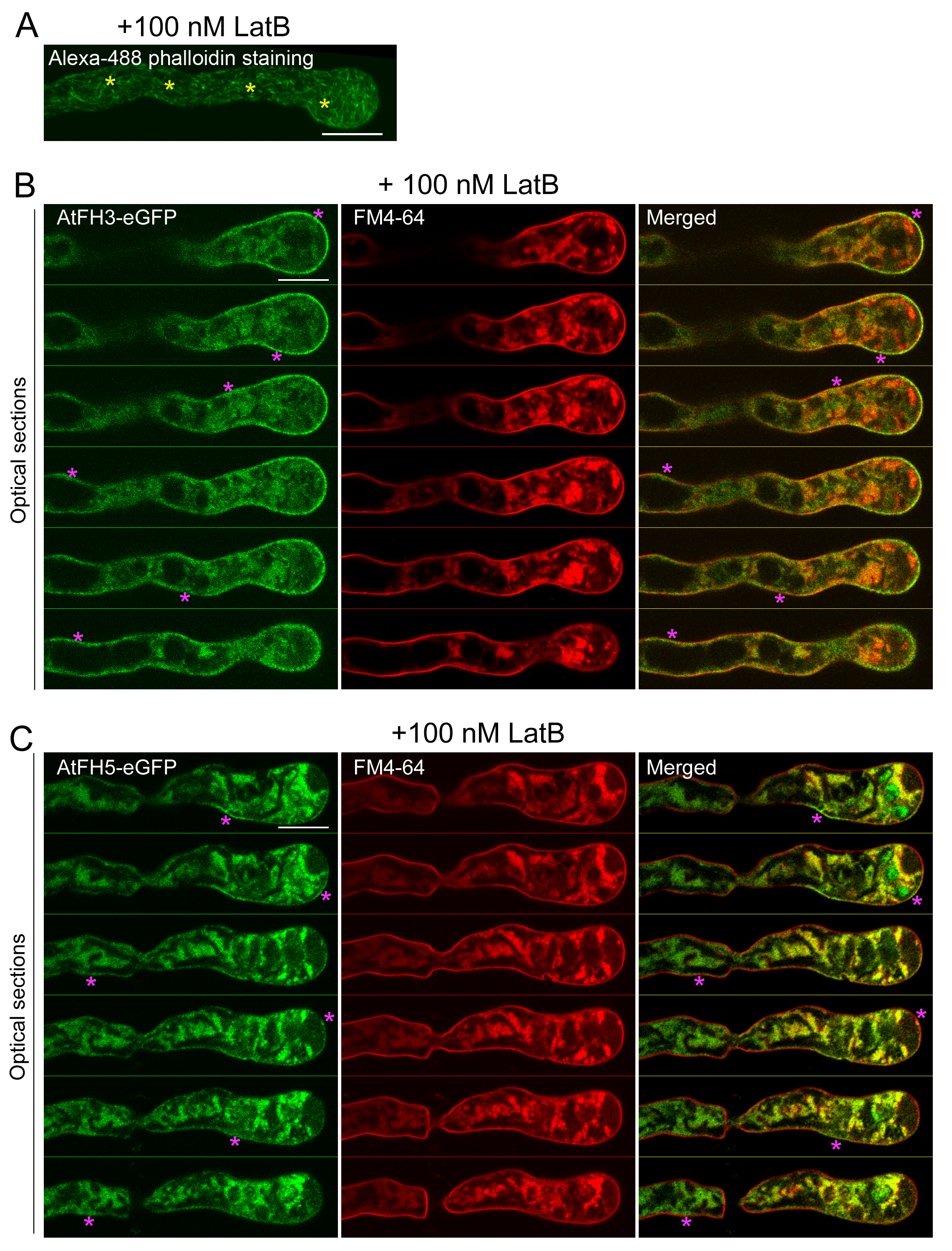

Supplement: S5 Fig — To determine whether the disruption of actin filaments affects PM targeting of AtFH3 and AtFH5, 100 nM latrunculin B (LatB) was applied onto the surface of solid pollen germination medium containing pollen for 30 min. Actin was then stained with Alexa488-phalloidin and pollen tubes were directly visualized with confocal microscopy. (A) Actin filaments stained with Alexa488-phalloidin in WT pollen tubes. Actin filaments are obviously depolymerized in WT pollen tubes after treatment with 100 nM LatB for 30 min. The projection image is presented and yellow asterisks indicate the disrupted actin filaments. Bar = 10 μm. (B) Distribution of AtFH3-eGFP in pollen tubes derived from AtFH3pro:FH3-eGFP;fh3-1 plants treated with 100 nM LatB. FM4-64 staining was employed to reveal the PM and endocytic vesicles. The magenta asterisks indicate the localization of AtFH3-eGFP on the PM. Bar = 10 μm. (C) Distribution of AtFH5-eGFP in pollen tubes derived from AtFH5pro:FH5-eGFP;fh5-2 plants treated with 100 nM LatB. FM4-64 staining was employed to reveal the PM and endocytic vesicles. The magenta asterisks indicate the localization of AtFH5-eGFP on the PM. Bar = 10 μm. (TIF) [file pgen.1007789.s005.tif]

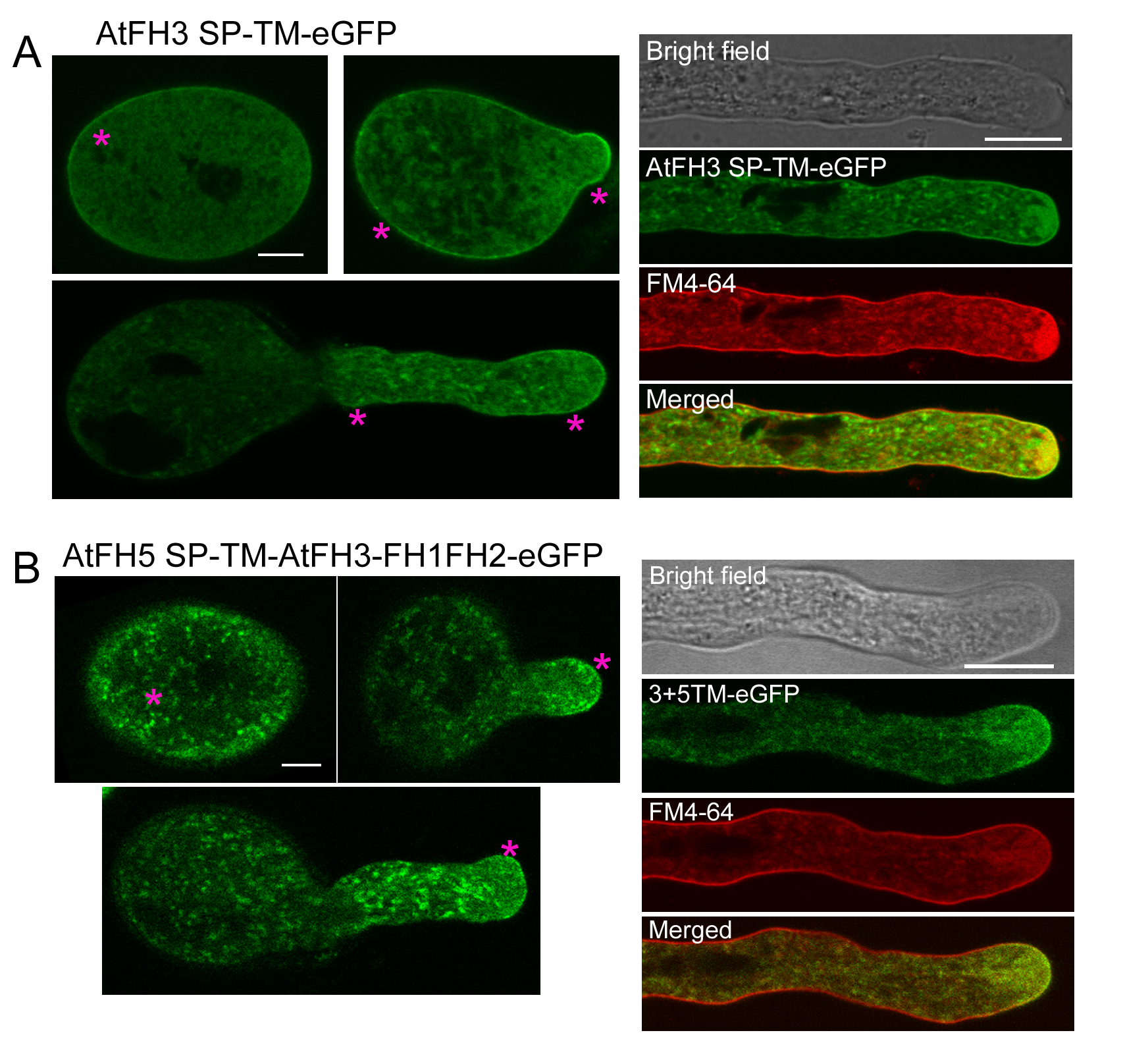

Supplement: S6 Fig — (A) Distribution of AtFH3-SP-TM-eGFP protein in ungerminated and germinated pollen derived from Arabidopsis plants harboring pCAMBIA1301-Lat52pro-AtFH3-SP-TM-eGFP. Medial optical sections are presented. The magenta asterisks indicate the localization of AtFH3-SP-TM-eGFP on the PM. Co-localization of AtFH3-SP-TM protein with FM4-64-stained plasma membrane and endocytic vesicles is presented in the right panel. Bars in left and right panels are 5 μm and 10 μm, respectively. (B) Distribution of AtFH5-SP-TM-AtFH3-FH1FH2-eGFP protein in ungerminated and germinated pollen derived from AtFH3pro-AtFH5-SP-TM-AtFH3-FH1FH2-eGFP;fh3-1 plants. Medial optical sections are presented. The magenta asterisks indicate the localization of the fusion protein on the PM. Co-localization of AtFH5-SP-TM-AtFH3-FH1FH2-eGFP protein with FM4-64-stained plasma membrane and endocytic vesicles is presented in the right panel. Bars in left and right panels are 5 μm and 10 μm, respectively. (TIF) [file pgen.1007789.s006.tif]

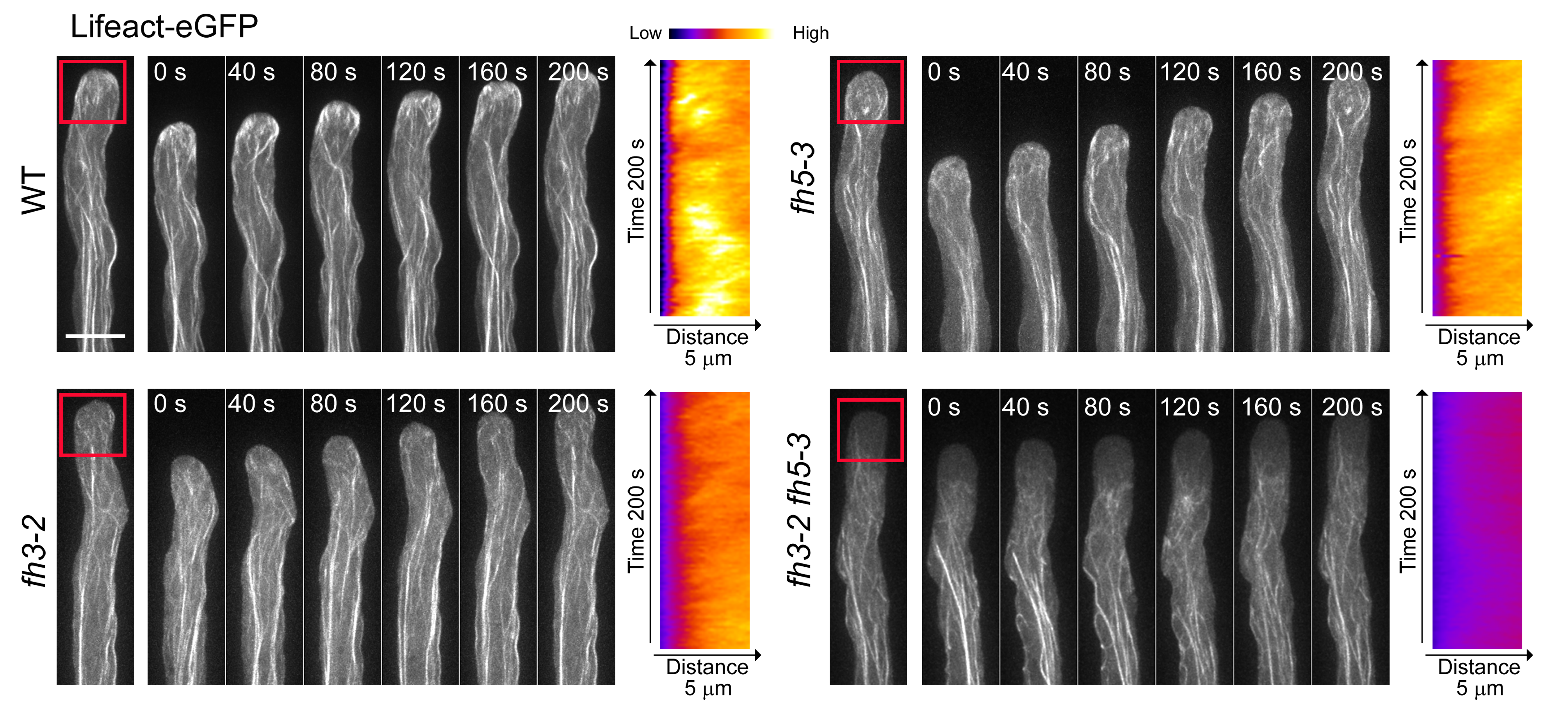

Supplement: S7 Fig — Time-lapse images of actin filaments revealed by decoration with Lifeact-eGFP in growing WT and fh3-2, fh5-3 and fh3-2 fh5-3 mutant pollen tubes. Red boxes indicate the apical region in pollen tubes. The right panels are kymograph images of the growing pollen tube tips after pseudo-color processing. Warm and cold colors indicate high and low fluorescence intensity, respectively. Bar = 10 μm. (TIF) [file pgen.1007789.s007.tif]
