# Supplementary material for: Arabidopsis class I formins control membrane-originated actin polymerization at pollen tube tips
Source: PLoS Genet. 2018 Nov 12;14(11):e1007789. doi: 10.1371/journal.pgen.1007789 (PMC6258422; doi:10.1371/journal.pgen.1007789)
Supplement: S1 Table — (DOCX) [file pgen.1007789.s018.docx]

**S1 Table. Primers used in this study**

| Name | Sequence (5’→3’) | Purpose |
| --- | --- | --- |
| *AFH3*pg-*Pst*I-F | CTGCAGCGACGCGGCTCAGCCAAG | To amplify the genomic sequence of *AtFH3* |
| *AFH3*pg-*Kpn*I-R | GGTACCGCCGCTGCCGCCCGAAGGTGAACTATCCTC |  |
| *AFH5*pro- F | TCTAGAGAGTTTTGGAATCAAGGTTTG | To amplify the promoter of *AFH5* |
| *AFH5*pro-R | CCCGGGATCTTTTGCTTCTCATTTCATCAGATACTCAATAG |  |
| *AFH5*genomic-F | CCCGGGATGGTTGGAATGATTCGAGGAGGAATGG | To amplify the genomic sequence of *AFH5* |
| *AFH5*genomic-R | GAGCTCGTCTGAATCTGAACTAGA |  |
| g*AFH5*-Mut-F | TCAAGCATAGATGATAAGAGTTCCAGATACTTGGGGTTATG | To amplify the *AFH5* genomic sequence with a disrupted *Sac*I restriction site |
| g*AFH5*-Mut-R | CATAACCCCAAGTATCTGGAACTCTTATCATCTATGCTTGA |  |
| eGFP*-Sac*I-F | GAGCTCATGGTGAGCAAGGGC GAGG | To amplify the *eGFP* coding sequence |
| eGFP*-Eco*RI-R | GAATTCTTACTTGTACAGCTCGTCCATG |  |
| *fh5-2* salk_044464-LP | AGCGTTTTTCTTAGCAGGAGG | Genotyping of *fh5-2* T-DNA insertion mutant |
| *fh5-2* salk_044464-RP | TGGTTGATTCTGTTTTCTGGG |  |
| *fh5-3* salk*_*152090-LP | TTTTCGATCAGGGTTGTTGAG | Genotyping of *fh5-3* T-DNA insertion mutant |
| *fh5-3* salk*_*152090-RP | AAGAGCTCCAGATACTTGGGG |  |
| *fh3-1* salk_150350-LP | AAGAAGCTCTCGGAACTCTCG | Genotyping of *fh3* T-DNA insertion mutant |
| *fh3-1* salk_150350-RP | TCTTCACATCTCGCAAAATCC |  |
| *fh3-2* CSHL_GT24923-LP | CCATCGAAATCTAAAAATGCG | Genotyping of *fh3-2* T-DNA insertion mutant |
| *fh3-2* CSHL_GT24923-RP | AAGAAGCTCTCGGAACTCTCG |  |
| Salk_LB 1.3 | ATTTTGCCGATTTCGGAAC | Genotyping of the T-DNA insertion lines |
| Ds3-1 | ACCCGACCGGATCGTATCGGT | Genotyping of *fh3-2* T-DNA insertion mutant |
| q-*eIF4A*-F | CGATGTGCAGCAAGTCTCTC | To amplify *eIF4A* as an internal loading control for qRT-PCR |
| q-*eIF4A*-R | CTCCCGAACCTTCCACTTCT |  |
| *AtFH3*-F1 | AAGATGTGGCAGAGCAAA | Used for qRT-PCR analysis to detect *AFH3* transcript levels |
| *AtFH3*-R1 | CAGGACCGAAAGCTAAATT |  |
| *AtFH3*-F2 | TAAGGAAACTACAAAGACGACG | Used for qRT-PCR analysis to detect *AFH3* transcript levels |
| *AtFH3*-R2 | AGCAATCGCTGGAAACAA |  |
| *AtFH5*-F | TTATGCTGCTCCAGGGTTT | Used for qRT-PCR analysis to detect *AFH5* transcript level |
| *AtFH5*-R | GCTTATCTCCTTTGACCGAAC |  |
| 3+5TM F1-*Xba*I | CTAGTCTAGACGACGCGGCTCAGCCAAG | Overlap PCR to amplify the genomic sequence of AtFH3pro-AtFH5SP-TM |
| 3+5TM R1-*Kpn*I | CGGGGTACCCGAAGGTGAACTATCCTCTTC |  |
| 3+5TM F2 | GAGAAACGCAAGTTGATGGTTGGAATGATT |  |
| 3+5TM R2 | CTTCAACGGAGGAAGAAGCCCGTGATTGGT |  |
| 3+5TM F3 | ACCAATCACGGGCTTCTTCCTCCGTTGAAG |  |
| 3+5TM R3 | AATCATTCCAACCATCAACTTGCGTTTCTC |  |
| 3+5TM-Mut-F | CAACAAAAACGTAGAAACTGCGGTCACAACAACAGCAATGATG | To amplify the AtFH3pro-AtFH5SP-TM genomic sequence with a disrupted *Pst*I restriction site |
| 3+5TM-Mut-R | CATCATTGCTGTTGTTGTGACCGCAGTTTCTACGTTTTTGTTG |  |
| 3+5TM F2-*Pst*I | AAAACTGCAGCGACGCGGCTCAGCCAAGTG | To amplify the AtFH3pro-AtFH5SP-TM genomic sequence |
| 3+5TM R2-*Xba*I | CTAGTCTAGAAAGCCCGTGATTGGT |  |
| AtFH3 FH1FH2-F | CTAGTCTAGACTTCCTCCGTTGAAGCTTCC | To amplify the AtFH3 FH1FH2 genomic sequence |
| AtFH3 FH1FH2-R | CGGGGTACCCGAAGGTGAACTATCCT |  |
| Lat52-F | CCCAAGCTTATACTCGACTCAGAAGGTAT | To amplify the promoter of *Lat52* |
| Lat52-R | AAAACTGCAGTTTTAAATTGGAATTTTTTTTTTTGGTGT |  |
| AtFH3-SPTM-F | AAAACTGCAGATGGGGAGATTGAGATTAGCG | To amplify the coding sequence of *AtFH3-SP-TM* |
| AtFH3-SPTM-R | CGGGGTACCTCCAGCTGCCGATGACG |  |
